# Comprehensive Biophysical Profiling Evidences Self‐Oligomerization of Bacterially Expressed Pc Protein

**DOI:** 10.1002/cbic.70405

**Published:** 2026-06-13

**Authors:** Maria Zahid, Sabin Prajapati, Ghazaleh Alamdari, Kai Tittmann, Selin Kara

**Affiliations:** ^1^ Institute of Technical Chemistry Leibniz University Hannover Hannover Germany; ^2^ Department of Molecular Enzymology Göttingen Centre for Molecular Biosciences (GZMB) and Albrecht‐von‐Haller‐Institute Georg‐August‐University Göttingen Germany; ^3^ Biocatalysis and Bioprocessing Group Department of Biological and Chemical Engineering Aarhus University Aarhus Denmark

**Keywords:** cryo‐electron microscopy (Cryo‐EM), intrinsically disordered protein (IDP), maltose‐binding protein (MBP), polycomb protein (Pc), self‐association/oligomerization

## Abstract

Polycomb (Pc) protein is a core component of Polycomb Repressive Complex 1 (PRC1) and plays a central role in epigenetic gene silencing, particularly during embryo development. Despite extensive functional characterization, structural understanding of Pc remains limited due to its intrinsic disorder, low sequence complexity, and propensity for dynamic self‐association. Here, we report the recombinant production, purification, and biophysical characterization of Drosophila melanogaster Pc constructs optimized for structural studies. Using solubility‐enhancing fusion strategies, we obtained milligram quantities of stable MBP‐tagged Pc protein suitable for downstream analyses. Sequence‐based disorder prediction, circular dichroism, and solution‐based techniques indicate that Pc is predominantly intrinsically disordered outside its conserved chromodomain, whereas it retains a defined secondary structure upon fusion and oligomerization. Thermal shift assays, Nano‐Differential Scanning Fluorimetry (nanoDSF), dynamic light scattering (DLS), and Size exclusion chromatography coupled to multi‐angle light scattering (SEC‐MALS) demonstrate strong buffer‐dependent stability and concentration‐dependent self‐association into higher‐order assemblies. Cryo‐electron microscopy reveals pronounced conformational and oligomeric heterogeneity, which can be partially stabilized by gradient fixation. Complementary AlphaFold‐based modeling supports a conditional folding mechanism in which Pc adopts more ordered architectures upon self‐association. Together, our results establish a robust framework for the biophysical investigation of Pc protein and provide mechanistic insight into how intrinsic disorder, oligomerization, and self‐association may contribute to Polycomb‐mediated chromatin regulation.

## Graphical abstract

Polycomb (Pc), a core component of Polycomb Repressive Complex 1 (PRC1), is largely intrinsically disordered yet forms buffer‐ and concentration‐dependent higher‐order assemblies. Integrative biophysical analysis and cryo‐electron microscopy reveal dynamic, heterogeneous oligomers, whereas AlphaFold modeling suggests that structural organization is conditional upon self‐association.

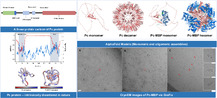

## 1 Introduction

The establishment and maintenance of cell identity during development require precise regulation of gene expression. In multicellular organisms, this regulation must be stably propagated through successive cell divisions despite changing cellular environments. In *Drosophila melanogaster*, as in other metazoans, this epigenetic memory is maintained in part by the coordinated action of Polycomb group (PcG) and Trithorax group (TrxG) proteins, which act antagonistically to repress or activate developmental gene programs, respectively. PcG proteins are essential for maintaining transcriptional repression of key developmental regulators, most notably the Homeotic (Hox) genes, whose misregulation leads to severe patterning defects and homeotic transformations [1].

PcG proteins exert their repressive functions through multiprotein assemblies known as Polycomb Repressive Complexes (PRCs). Among these, Polycomb Repressive Complex 1 (PRC1) plays a central role in chromatin compaction and transcriptional silencing [2]. The canonical PRC1 complex in *Drosophila* consists of Polycomb (Pc), Polyhomeotic (Ph), Posterior Sex Combs (Psc), and dRING1. It is recruited to chromatin in part through recognition of the histone H3 lysine 27 trimethylation (H3K27me3) mark deposited by PRC2 [3, 4, 5]. Pc, a founding member of the PcG family, functions as a key reader of this repressive mark via its N‐terminal chromodomain [6, 7]. In addition, its C‐terminal region mediates protein–protein interactions essential for PRC1 assembly and function [8, 9]. Various studies have demonstrated that PRC1 also binds to promoter sites with low or no H3K27me3, suggesting that PRC1 can be targeted independently of the Pc chromodomain [10], underscoring the need for deeper study of the structural dynamics of other domains.

The *Pc* gene encodes a 390 amino acid (aa) protein (≈44 kDa) that is highly enriched in charged residues, rendering it hydrophilic and low in sequence complexity. Pc contains two conserved structured elements, an N‐terminal **chromodomain** and a C‐terminal **C‐box**, flanked by extended regions of low complexity and intrinsic disorder [11]. The Chromodomain comprises 50–60 amino acids and contains one α‐helix and three β‐sheets, forming a cleft where the histone H3 peptide interacts by recognizing the K27me3 mark [6, 7]. Pc interacts with its targets on the chromatin by binding to the specific loci via its chromodomain. Chromodomains are present in many proteins, most of which are associated with compact, inactive chromatin, i.e., silenced genes [12, 13]. Structural studies have revealed that the CBX7 Cbox domain engages C‐RING1B through an extensive and highly specific interface, centered on the formation of an intermolecular antiparallel β‐sheet. This interaction is further stabilized by complementary hydrophobic packing and polar contacts involving conserved aromatic, aliphatic, and charged residues, underscoring the structural sophistication underlying CBX7‐RING1B complex formation [14, 15].

In addition, Pc harbors two conserved **histidine‐rich motifs** (8 and 10 residues long), 4 nuclear localization signals, and a central region implicated in chromatin compaction and phase separation (CaPS) [16]. The function of His‐rich motifs remains incompletely understood; however, His‐rich peptides are generally known for their role in intracellular trafficking, specifically enhancing endosomal escape via osmotic swelling [17].

Despite extensive genetic and biochemical characterization, the molecular mechanisms by which Pc contributes to transcriptional repression remain incompletely understood. While the chromodomain has been structurally resolved, accumulating evidence indicates that Pc‐mediated repression is not solely dependent on chromodomain function. Pc can repress transcription even in the absence of a functional chromodomain when artificially tethered to DNA [18], suggesting that additional regions play critical roles in gene silencing. In particular, the C‐terminal region of Pc interacts with core PRC1 components, including RING1 proteins and Psc, implicating this region in chromatin compaction and maintenance of the repressed state [8, 14].

Pc protein can also contribute to transcriptional repression through noncanonical mechanisms. Pc directly inhibits histone acetylation by binding to the auto‐inhibitory loop of the histone acetyltransferase CREB‐binding protein (CBP) via a conserved **KRG motif** located immediately downstream of the chromodomain [19, 20]. The KRG motif in the Pc protein is a short and conserved segment with the core sequence Lys‐Arg‐Gly. This interaction via the KRG motif interferes with CBP autoacetylation and activation, thereby suppressing transcriptional activation. Pc and CBP co‐occupy promoters associated with paused RNA polymerase II, indicating a direct role for Pc in regulating transcriptional dynamics independent of PRC2‐mediated H3K27 methylation [21]. ChIP‐seq and RNA‐seq‐based genome‐wide studies reveal that, in general, Pc preferentially binds to genomic regions encoding transcription factors, as well as to genes involved in signaling and developmental pathways [22, 23]. Recently, it was found that C‐terminal binding protein (CtBP) plays a direct role in the “molecular switching” between H3K27me3 and H3K27ac [24]. This interaction highlights a potential noncanonical mechanism by which Pc contributes to transcriptional repression, independent of PRC2‐mediated histone methylation.

Many of the seminal studies conducted over the past two decades have significantly contributed to our current understanding of the structure and functional roles of PcG proteins. The crystal structure of PRC1 bound to the nucleosome shows that one copy of the PRC1 ubiquitylation module (without the Pc protein) forms a crescent shape with E2 and E3 ubiquitin ligases and binds to the disk face of the nucleosome [25]. A recent study by Ciapponi et al. provided further insights into the PRC1 complex, although excluding the Pc (CBX) protein. In this minimal PRC1 complex, the mechanism of histone H2A monoubiquitination and its interaction with the RYBP protein were studied. However, the density of any of the proteins in the complex, namely, RING1B, BMI1, or RYBP, could not be measured [26].

A recurring theme in studies of Pc and its vertebrate homologs (CBX2, 4, 6, 7, and 8) is that they are intrinsically or partially disordered proteins that can form higher‐order assemblies and biomolecular condensates within the nucleus [27, 28]. Outside the chromodomain and C‐box, Pc is largely unstructured, which suggests that Pc function may depend on context‐dependent structural organization rather than a single rigid conformation. Our preliminary NMR experiments (Figure S2) and prediction models (Figure 6) coherently show that Pc is an intrinsically disordered protein (IDP). IDPs play essential roles in several cellular processes, ranging from transcription factors to structural proteins [29]. Such IDRs are increasingly recognized as key drivers of multivalent interactions, dynamic assemblies, and the formation of biomolecular condensates in the nucleus [30]. The standard structural tools are not yet mature for IDPs, but significant progress has been made in the last decade. In this context, CryoEM, along with other structural techniques, has proven extremely useful [31]. Moreover, computational tools have greatly assisted this understanding [32].

In the nucleus Pc, along with other PcG proteins, localizes to discrete foci in the form of condensates known as Polycomb group (PcG) bodies, which display dynamic distribution and variable intensity during embryogenesis. Briefly, biomolecular condensates are defined as nonstoichiometric assemblies of biomolecules that can contain multiple components [33]. The PcG bodies share hallmarks of phase‐separated assemblies, including sensitivity to protein concentration, dependence on electrostatic interactions, and dynamic component exchange [28]. The dynamic distribution of PcG bodies is not fully understood; however, they serve as reservoirs for member proteins in nuclear speckles and provide a platform for interaction with Polycomb response elements (PREs), thereby facilitating gene silencing [34, 35]. In mammalian PRC1, phase separation is driven by CBX2 via a positively charged low‐complexity disordered region (LCDR), with the C‐box domain playing a critical role in condensate integrity [36]. These condensates are formed via a scaffold‐client mechanism, in which CBX2 acts as the scaffold. CBX2 can form single‐component condensates at concentrations as low as 10 nM, which are facilitated by the presence of Mg^2^ ions [28]. In a seminal work, Guan et al. recently showed that the mutated CBX6, where the C‐box domain is missing, fails to assemble into the Pc bodies and leads to malfunctioning condensates [37].

Across the recent work, a coherent picture has emerged in which Pc proteins act as both drivers and regulators of biomolecular condensates that modulate gene repression. Early evidence from the CBX2 study established that the Pc homolog CBX2 possesses an intrinsically disordered, positively charged region that enables it to phase separate into liquid‐like nuclear condensates, providing a biophysical basis for Polycomb body formation [38]. Building on this, Brown et al. demonstrated that condensate formation is cooperative and sequence‐encoded, with charge patterning in CBX2's disordered region governing assembly and phosphorylation, offering a mechanism for dynamic regulation [28]. More work from Niekamp et al. refines this model by showing that PRC1 condensates arise from multivalent interactions distributed across subunits and strongly enhanced by chromatin, positioning Pc as part of a broader interaction network rather than the sole structural driver [39]. Very recently, Ingersoll et al. proposed a nucleation‐ and bridging‐induced phase separation (NBiPS) model, revealing that small clusters of murine CBX2 can self‐associate to seed larger Polycomb assemblies, which subsequently grow through recruitment of additional complexes and chromatin engagement [40]. Together, these studies support a unified model in which Pc proteins (Drosophila Pc and its homologs) exhibit intrinsic self‐association capacity that is amplified by chromatin and partner proteins, enabling the nucleation, growth, and regulation of dynamic PRC1 condensates that organize repressive chromatin architecture.

AI‐based structure prediction tools, such as AlphaFold (AF) have provided new insights into proteins that are challenging to characterize using classical structural approaches. AF models predict that Pc is predominantly disordered outside the chromodomain, consistent with its sequence composition and biophysical properties. More specifically, advances in AF‐based methods have significantly extended their applicability from monomeric structure prediction to protein complex and assembly inference. In particular, AF‐Multimer has demonstrated that protein–protein interfaces can be predicted with high accuracy [41]. This approach successfully captures interaction geometries for both homomeric and heteromeric assemblies, indicating that sequence information encodes sufficient constraints to reconstruct many biologically relevant protein–protein interfaces.

Along these lines, a recent work from Lin et al. has further evaluated whether AF outputs can be interpreted beyond structure prediction to infer protein oligomeric states [42]. Specifically, systematic benchmarking shows that both AF‐Multimer and AF3, predictions often correlate strongly with experimentally observed assembly states. A key observation is that interface confidence metrics, particularly ipTM (interface predicted TM‐score), provide a quantitative indicator of whether a predicted complex reflects a biologically relevant oligomer or an artefactual association. Together, these studies indicate that AF‐based models are not only structure‐predicting tools but also implicitly learn rules governing protein assembly and quaternary structure formation. However, there are still major limitations to be considered. The computational costs for high(er) oligomeric states are quite large, and the performance decreases for transient, weak, or context‐dependent interactions, indicating that current models can primarily capture only stable equilibrium assemblies.

As mentioned earlier, structural information on Pc protein remains limited. While high‐resolution structures have been obtained for individual PRC1 subcomplexes and ubiquitination modules, Pc (other than the Chromodomain) has largely been excluded from these studies, in part due to challenges associated with its intrinsic disorder, poor solubility, and conformational heterogeneity [4]. Advances in cryo‐electron microscopy (cryo‐EM) and integrative structural approaches now offer new opportunities to investigate such proteins, provided that sufficient quantities of stable and homogeneous material can be obtained. In this study, we focus on the recombinant production, purification, and biophysical characterization of *Drosophila melanogaster* Pc protein constructs optimized for structural analysis. By employing solubility‐enhancing fusion strategies and systematically optimizing bacterial expression and purification conditions, we aim to generate the Pc protein suitable for downstream cryo‐EM analysis. By combining cryo‐EM with AI‐based modeling, we investigate Pc self‐association and propose a framework linking intrinsic disorder, conditional folding, and higher‐order assembly in the context of Pc condensate formation. Our work establishes a foundation for future structural studies of Pc and provides mechanistic insight into how structural plasticity contributes to Polycomb‐mediated gene silencing.

## 2 Materials and Methods

The detailed methods for protein expression and purification are provided in the Supplementary Information file. The MBP tag was chosen as a fusion partner for enhanced solubility and correct folding. Briefly, Pc‐MBP constructs were designed for bacterial expression [43, 44]. Two *E. coli* strains, BL21 Rosetta and Shuffle T7, were used for expression trials. Proteins were purified by immobilized metal affinity chromatography (IMAC), followed by MBP‐Trap affinity chromatography, and finally by size‐exclusion chromatography for polishing and buffer exchange.

### 2.1 Nano‐Differential Scanning Fluorimetry (NanoDSF)

Protein thermal stability was assessed by nanoDSF using Prometheus NT.Plex instrument (NanoTemper GmbH). Purified Pc‐MBP‐SHuffle T7 and Pc‐MBP‐Rosetta 2 proteins were analyzed in multiple buffer systems, including Tris‐HCl, Bis‐Tris, MOPS, HEPES, phosphate, acetate, and MES/NaOH (buffer compositions listed in SI), covering a pH range from 4.0 to 8.0. Protein samples expressed in *E. coli* Rosetta 2 were prepared by diluting 14 µL of protein stock solution with 86 µL of the respective buffer, whereas samples expressed in *E. coli* SHuffle T7 were prepared by diluting 12.5 µL of protein stock solution with 87.5 µL of buffer, resulting in a final protein concentration of 0.2 mg/mL. Samples were incubated on ice for 1 h to ensure homogenization before analysis. NanoDSF measurements were performed over a temperature range of 25°C–95°C. Buffer solutions were measured as blanks before sample analysis. For each condition, three capillaries were filled from the same reaction mixture to obtain technical triplicates, and the capillaries were inserted into the instrument for simultaneous measurement. Intrinsic fluorescence at 330 and 350 nm was recorded during the thermal ramp, and melting temperatures (Tm) were determined from the fluorescence ratio (F350/F330) using the instrument's analysis software.

### 2.2 Dynamic Light Scattering (DLS) and Multi‐Angle Light Scattering (MALS) Measurements

The hydrodynamic radius and size distribution of protein samples were determined by DLS. Recombinant Pc‐MBP‐SHuffle T7 and Pc‐MBP‐Rosetta 2 proteins were analyzed in different storage buffers, including Tris‐HCl and HEPES. Concentrated protein stocks of Pc‐MBP‐SHuffle T7 and Pc‐MBP‐Rosetta 2 were diluted to a final concentration of 0.2 mg/mL in either 50 mM Tris‐HCl (pH 8.0) or 50 mM HEPES (pH 8.0). All samples were prepared in duplicate to ensure reproducibility and were gently mixed before analysis. The diluted protein solutions were transferred into 11 mm UVette low‐volume plastic cuvettes and analyzed using a DLS spectrophotometer (LitesizerTM 100, Anton Paar GmbH). Measurements were performed at a controlled temperature of 25°C following an equilibration period of 2 min. The instrument software was operated in automatic mode, with automatic optimization of the scattering angle based on sample transmittance, automatic adjustment of optical filter density and focus position, and a general analysis model suitable for samples with unknown size distributions. Each measurement consisted of 10‐s acquisition runs, with up to 60 runs recorded per sample. The hydrodynamic radius values were calculated from the averaged correlation functions generated by the instrument software, Kalliope (Anton Paar GmbH).

For MALS analysis, size‐exclusion chromatography coupled to multi‐angle light scattering (miniDAWN TREOS, Wyatt Technology Europe) was used to determine the absolute molecular weight and the protein's oligomeric state in solution. 300 μL of the protein samples (concentration 1 mg/mL) were injected onto a Superose 6 Increase column (Cytiva Europe GmbH) equilibrated with 50 mM Tris‐HCl buffer, pH 8.0, and eluted at a constant flow rate (0.5 mL/min). Light scattering and refractive index signals were recorded, and molecular weights were calculated using standard protein refractive index increment (dn/dc) values. Data were analyzed using the corresponding analysis software, ASTRA (Wyatt Technology Europe).

### 2.3 Circular Dichroism (CD) Measurements

Protein secondary structure was analyzed by CD spectroscopy. Pc‐MBP protein samples were prepared by diluting protein stock solution (0.5 mg/mL) in 300 µL of 50 mM Tris‐HCl buffer, pH 8.0, resulting in a final protein concentration of 1.67 mg/mL. CD measurements were performed at room temperature using a Jasco J‐810 spectropolarimeter equipped with a 1 mm path length quartz cell. Spectra were recorded between 190 and 260 nm with a 1 nm bandwidth, 1 s response time, and a scan speed of 50 nm/min. Three scans were acquired per sample and averaged, and buffer spectra were subtracted from the protein spectra to correct for baseline contributions. Data were expressed as mean residue ellipticity (MRE) and analyzed using the BeStSel algorithm, which is optimized for deconvolving β‐sheet‐rich protein structures [45].

### 2.4 Sample Preparation via the GraFix Method and Grid Preparation

The protein sample was stabilized using the gradient fixation method [46]. Shortly, 300 mg/mL Pc‐MBP was loaded on top of the 10–30% linear sucrose gradient made in SW60 ultracentrifugation tubes using Gradient Master 108 (BIOCOMP). The sucrose solution was prepared in a 50 mM HEPES, 200 mM NaCl, pH 8.0 buffer that additionally contained 5 mM DTT and 1 mM EDTA. For GraFix, the 30% sucrose stock also contained 0.1% w/v glutaraldehyde to create a 0–0.1% glutaraldehyde gradient. Ultracentrifugation was performed for 16 h at 25,000 × g and 4°C, and the fractions were collected manually from the top using a micropipette. A 50 mM Tris‐HCl, pH 8 buffer was added to the fractions to quench the unreacted glutaraldehyde. To identify the fractions containing the protein, 1 µL of solution from each fraction was loaded onto nitrocellulose paper. After adsorption, it was stained for 2 min with a 0.1% amido black solution. The de‐staining was performed using water. The protein‐containing fractions, as identified by the Dot‐Blot, were pooled, and sucrose was removed using a centrifugal protein concentrator (30 kDa molecular cutoff, Amicon).

#### 2.4.1 Data Collection

A 3‐μl +/− GraFixed MBP‐PC (0.2 mg/mL) sample was blotted on glow‐discharged Quantifoil Cu R2/1, 200 mesh grids. Plunge‐freezing was performed with a Vitrobot Mark IV (Thermo Fisher Scientific) in a chamber equilibrated at 10°C with 100% humidity. Cryo‐EM data were collected on a Titan Krios G4 transmission electron microscope (Thermo Fisher Scientific) operated at 300 kV and equipped with a Selectris energy filter (10 eV slit width) and a Falcon 4 direct electron detector. Images were recorded using EPU software at a nominal magnification of 165,000×, corresponding to a calibrated physical pixel size of 0.72 Å. Movies were collected with a total electron dose of 38 e^−^/Å^−2^ and a defocus range of 1.5 to −2.5 µm.

#### 2.4.2 Data Analysis

We used CryoSparc v4.6.2 for motion correction and CTF estimation. Micrographs were denoised using the cryoSPARC micrograph denoising tool before particle picking [47]. However, owing to a very high degree of sample heterogeneity, we could not calculate 2D‐class averages. So, we manually selected the micrographs containing intact particles and fewer aggregations to compare +/− GraFix samples.

### 2.5 Structure Prediction Models via AF3

The structural models for monomeric and multimeric forms were predicted using AF3 via the AF server [48]. The amino acid sequences of Pc full‐length (wildtype and N‐terminal MBP‐fused) were used as input. The predicted models were selected based on the highest pLDDT scores. To model oligomeric assemblies, multiple copies of the same sequence were used as the input in a single run, corresponding to the desired stoichiometry (monomer, dimer, trimer, tetramer, hexamer, and decamer). The structure of the higher oligomeric state could not be predicted due to the computational restrictions. Default AF3 settings were used unless otherwise specified, and no symmetry constraints were explicitly imposed. For each oligomeric state, multiple prediction runs were performed, and the top‐ranked models were selected based on the model confidence metrics provided by AF3 (including predicted alignment error and per‐residue confidence scores). Models were inspected in PyMol to ensure consistency across runs.

The radii of gyration were calculated from the principal axis of protein models in ChimeraX using Equation (1).

$$R_{g}≅\frac{\sqrt{r_{1}^{2}+r_{2}^{2}+r_{3}^{2}}}{3}$$

All structural visualizations and color‐coding of domains were performed in PyMol with consistent coloring applied across all oligomeric states to facilitate comparison. The maximum‐pairwise distances of the AF oligomer models were calculated using PyMol and ChimeraX, and were used for structure visualization [49, 50].

## 3 Results and Discussion

### 3.1 Secondary and Tertiary Structure Analysis

ProtParam analysis predicts a molecular weight of 43.98 kDa (pI 7.10) for Pc and 87.35 kDa (pI 6.25) for the Pc‐MBP fusion protein, which are consistent with SDS‐PAGE observations. ProtParam analysis further indicates that both Pc and Pc–MBP exhibit negative GRAVY scores (−1.231 and −0.803, respectively), suggesting an overall hydrophilic character. Sequence‐based disorder predictions consistently identified extended regions of intrinsic disorder within the Pc protein. These regions are enriched in low‐complexity residues and lack predicted secondary structure, a feature commonly observed in regulatory and interaction‐prone proteins. Sequence analysis revealed the presence of LCDRs enriched in Serine (aa177–200) and Glutamine (aa236–254) spanning the C‐terminal domain. While this region shows limited sequence conservation, its compositional bias and positional conservation suggest a potential role in protein–protein interactions or regulatory processes. Similar low‐complexity regions have been reported in other protein classes, such as chromatin‐associated proteins (CAPs), where they regulate PTMs and protein–protein interaction modules [31, 51]. Consistent with these predictions, AF models showed low per‐residue confidence scores in the same regions, suggesting the absence of a stable folded structure in the monomeric state. Such convergence between sequence‐based and structure‐based analyses supports the presence of intrinsically disordered regions rather than artifacts of individual prediction methods.

**FIGURE 1:**
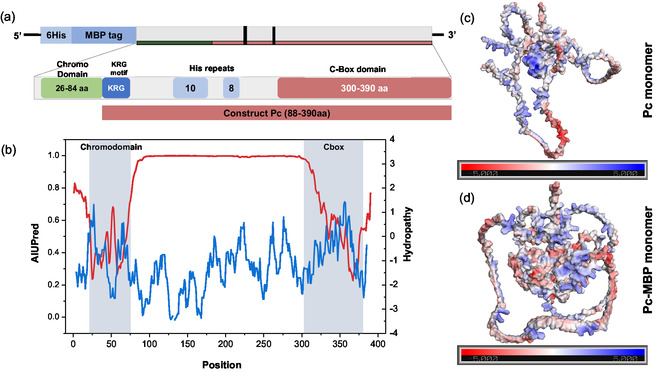
Graphical representation of the Pc protein. (a) The 390 aa‐long protein comprises an N‐terminal chromodomain, a short KRG (Lys‐Arg‐Gly) motif, two histidine stretches (10× & 8×), and a C‐terminal C‐box domain. The lower panel (b) shows the disorder score in red (predicted via AIUPred) and the Hydropathy score in blue, calculated according to the Kyte–Doolittle method via Expasy Hydropathy plot. Electrostatic surface potential maps of Pc in the absence (c) and presence (d) of an N‐terminal MBP tag (with more structured core), calculated using APBS in PyMOL and mapped onto AF3‐predicted structures. Surface potentials are displayed on a scale from −5 kT/e (red, negatively charged) to +5 kT/e (blue, positively charged).

To further characterize the physicochemical properties of the Pc protein, we analyzed the electrostatic surface potential of the protein with and without the MBP tag (Figure 1). The untagged Pc protein displays a dispersed electrostatic profile consistent with its intrinsically disordered nature (Figure 1c), whereas the MBP‐tagged construct adopts a more compact conformation (with noticeable structured core, also seen in the AF models in Figure 6) with distinct regions of concentrated positive and negative charge (Figure 1d). These changes indicate that the MBP fusion substantially alters the electrostatic landscape of the Pc protein. Given the established role of electrostatic interactions in mediating protein–protein interactions and condensate formation, such differences are likely to influence Pc self‐association and higher‐order assembly. In particular, the redistribution and clustering of charged regions in the MBP‐tagged construct may affect interaction interfaces and modulate assembly propensity. While these observations are based on predicted structures and should therefore be interpreted with caution, they provide additional insight into how fusion tags may impact the biophysical behavior of IDPs.

Intrinsically disordered regions are frequently involved in transient interactions and oligomerization processes, where structural plasticity enables adaptive binding to multiple partners or conformational states. This is evident from the predictive models of oligomeric forms, as shown in Figure 6. Although the Pc‐MBP model is partially disordered in the monomeric state, self‐association into higher‐order oligomers is likely to promote structural ordering. Intrinsically disordered regions are known to undergo disorder‐to‐order transitions upon binding or oligomerization, a process often described as coupled folding and assembly. In this context, multimer formation can stabilize specific conformations through intermolecular contacts, thereby reducing conformational heterogeneity and increasing overall structural order [38].

### 3.2 Recombinant Expression of Pc Protein in the Bacterial System

Five constructs of the Pc protein, including a full‐length design, were generated for bacterial expression. Based on the highest expression levels, PcC1 (hereafter Pc) was selected for further studies. The production of Pc‐MBP was evaluated in *E. coli* Rosetta 2 and SHuffle T7 strains. SDS‐PAGE analysis confirmed expression of the 88 kDa Pc‐MBP protein in both hosts. In Rosetta 2, faint bands were detected before induction, indicating leaky expression characteristic of the T7 promoter, whereas SHuffle T7 showed no pre‐induction expression, demonstrating tighter transcriptional control. Optimization of induction conditions revealed that IPTG concentration influenced solubility. In SHuffle T7, 0.25 mM IPTG yielded higher levels of soluble protein and reduced accumulation of insoluble aggregates compared to 0.5 mM IPTG (data not shown).

**FIGURE 2:**
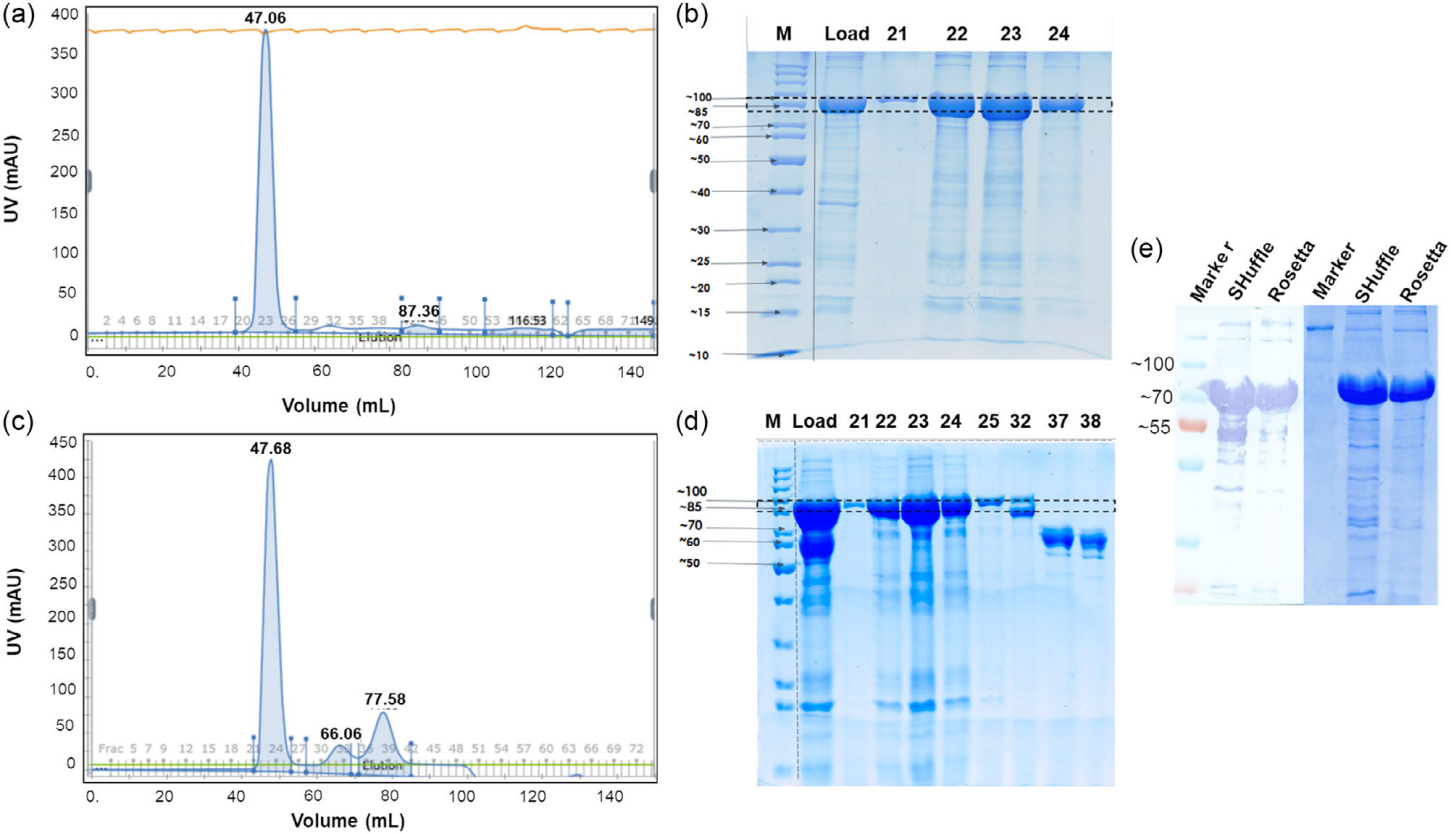
Size‐exclusion chromatography (SEC) of Pc‐MBP‐Rosetta (a), Pc‐MBP‐Shuffle, and the corresponding (c) and SDS‐PAGE analyses (b & d). Prior to SEC, the proteins were purified via His‐tag and MBP‐tag affinity chromatography steps. SEC of and SDS‐PAGE (d) of elution fractions. The elution fractions were pooled, concentrated, and analyzed by SDS‐PAGE (e, right panel) and Western blotting using anti‐His antibody (e, left panel).

Purification of Pc‐MBP was achieved through IMAC, MBP‐Trap, and size exclusion chromatography (SEC). IMAC purification showed partial protein loss in the flow‐through, likely due to misfolding or suboptimal resin binding. MBP‐Trap efficiently captured the target protein, with elution fractions displaying the highest concentrations. SEC confirmed protein homogeneity, with the major peak for Rosetta observed in fractions 22–23 and for SHuffle T7 in fractions 23–24, both at a retention volume of 47 mL (Figure 2). SDS‐PAGE and Western blot analysis with an anti‐His antibody confirmed the protein's identity and purity in both strains (Figure 2). Quantitative analysis revealed final yields of pure Pc‐MBP of 7.23 mg/L for Rosetta and 5.68 mg/L for SHuffle. The higher yield in Rosetta 2 likely reflects enhanced codon adaptation and tRNA availability, whereas SHuffle T7, engineered for cytoplasmic disulfide bond formation via constitutive DsbC expression, provided well‐folded protein with reasonable solubility despite slightly lower yields.

Following removal of the MBP tag, Pc formed insoluble aggregates that could not be readily resolubilized using urea‐ or guanidine hydrochloride‐based approaches (For details, see Supplementary Information). This behavior is consistent with the strong solubilizing effect of MBP on aggregation‐prone proteins and suggests an intrinsic tendency of Pc to aggregate during heterologous expression in *E. coli* [52]. Therefore, all further analyses were performed with the MBP‐Pc fusion protein.

### 3.3 Thermal Stability of Protein

The effect of buffer on protein stability has been very well‐established, both empirically and computationally [53]. To identify stabilizing buffer conditions for truncated Pc constructs, a thermal shift assay (TSA) was performed in a microtiter plate (MTP) format using SYPRO Orange as an extrinsic fluorescent probe. This approach enables rapid and parallel evaluation of protein thermal stability across a broad range of solution conditions. The resulting melting temperatures (Tm), summarized in the heatmap (Figure S3), provide a comparative overview of protein stability across the tested buffer conditions. Overall, most of the buffer conditions yielded Tm values in the range of approximately 44°C–48°C, indicating moderate intrinsic stability of the truncated PcC1 construct. However, several buffer conditions produced markedly higher Tm values, reaching up to ∼55°C–56°C, suggesting a significant stabilizing effect. These buffers, namely Tris‐HCl, HEPES, and MOPS, are clearly distinguishable in the heatmap by their red coloration and represent the most promising candidates for downstream biochemical and structural studies. A subset of conditions yielded lower apparent melting temperatures (∼37°C–40°C) or no detectable transition (“nd”), likely due to partial unfolding, aggregation, or insufficient SYPRO Orange binding, and were therefore excluded from further analysis (Figure S3b).

**FIGURE 3:**
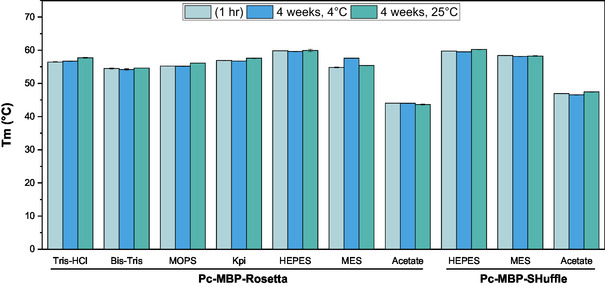
Thermal stability of Pc‐MBP in different buffer systems using NanoDSF. The melting temperatures (Tm) of the Pc protein were measured after 1 h at 4°C (cyan) and after storage at 4°C (blue) and 25°C (green) for 4 weeks. Samples were stored in the indicated buffer systems (HEPES, Tris‐HCl, KPi, MOPS, MES, Bis‐Tris, and Acetate), and the Tm measurements were performed at least in duplicate.

To further assess the thermal stability of Pc‐MBP under selected buffer conditions, NanoDSF was used after storage at 4°C or 25°C. Changes in the intrinsic fluorescence ratio (F350/F330) revealed buffer‐dependent differences in protein stability and unfolding behavior (Figure 3 & S4). Immediately after preparation (1 h at 4°C), Pc‐MBP exhibited a clear and cooperative thermal unfolding transition in HEPES, Tris‐HCl, KPi, and MOPS buffers, with apparent melting temperatures (Tm) of approximately 60–70°C. In contrast, MES and acetate buffers showed lower baseline fluorescence ratios and less pronounced unfolding transitions, indicating reduced structural stability or partial destabilization of the protein even under fresh conditions (Figures 3 & S4).

Following 4 weeks of storage at 4°C, Pc‐MBP largely retained its thermal stability in HEPES, Tris‐HCl, KPi, and MOPS buffers, although a slight decrease in Tm and reduced cooperativity of unfolding were observed. The first‐derivative curves still displayed distinct minima, consistent with a predominantly folded protein population. In contrast, samples stored in MES and acetate buffers showed weakened or broadened transitions, suggesting increased conformational heterogeneity, partial unfolding, or aggregation during cold storage.

Storage at 25°C for 4 weeks resulted in a pronounced loss of thermal stability across most of the buffer conditions. While Pc‐MBP in HEPES, Tris‐HCl, and KPi buffers still exhibited detectable unfolding transitions, these were shifted to lower temperatures and displayed reduced cooperativity, indicating significant structural destabilization. In MES and acetate buffers, unfolding transitions were largely absent, consistent with severe destabilization or irreversible structural damage. Overall, these results demonstrate that Pc‐MBP stability is strongly buffer‐dependent, with HEPES and Tris‐HCl providing the most robust protection against thermal and storage‐induced denaturation, whereas MES and acetate are unsuitable for maintaining protein integrity.

#### 3.3.1 DLS Measurement for Size Analysis

To assess the solubility and homogeneity of the purified protein, DLS measurements were performed at 25°C for 1 h and 4 weeks. DLS measurements revealed pronounced differences in the particle‐size distributions of the Pc‐MBP fusion proteins, depending on the expression strain and buffer composition (Figure S5). Pc‐MBP produced in Shuffle T7 cells in Tris‐HCl displayed the smallest Z‐average hydrodynamic diameter (∼269 nm), whereas the same protein in HEPES formed much larger species, indicating pronounced aggregation (Table 1). Rosetta‐derived samples exhibited even greater apparent sizes in both buffers (≈1–10 µm range), consistent with extensive higher‐order assembly.

**TABLE 1:** DLS analysis of Pc‐MBP fusion proteins produced in *E. coli* Shuffle T7 and Rosetta 2 strains in different buffer systems. Shown are the Z‐average hydrodynamic diameter (Hyd), polydispersity index (PDI), and intensity‐weighted size distribution peaks (Peaks 1–3) for samples in Tris‐HCl and HEPES buffers. Multiple peaks indicate heterogeneous particle populations and the presence of higher‐order species.

Buffer	Pc‐MBP‐SHuffle T7					Pc‐MBP‐Rosetta 2				
Tris‐HCl	Hyd	PDI	Peak1	Peak 2	Peak 3	Hyd	PDI	Peak1	Peak 2	Peak 3
	269.1 nm	27.10%	88.1 nm	1268.3 nm	—	1026.3 nm	28.90%	82.9 nm	1085.8 nm	6986.5 nm
HEPES	Hyd	PDI	Peak1	Peak 2	Peak 3	Hyd	PDI	Peak1	Peak 2	Peak 3
	155062 nm	26.80%	45.8nm	122.6 nm	—	144122 nm	20.10%	43.0 nm	195.8 nm	314.8 nm

**FIGURE 4:**
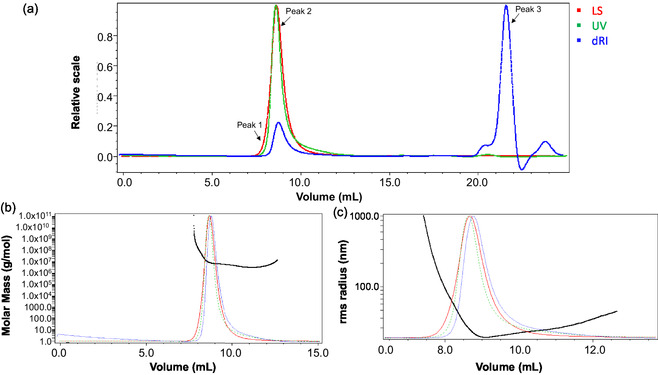
SEC‐MALS analysis of Pc‐MBP in 50 mM Tris‐HCl, 200 mM NaCl, 1 mM DTT, pH 8.0. (a) SEC‐MALS showing the elution profile of the Pc protein monitored by light scattering (LS, red), UV absorbance at 280 nm (UV, green), and differential refractive index (dRI, blue). The molar mass (b), polydispersity, and rms radius moments (c) of the main population in peak 2 are shown in the lower panel as well as given in Table 2. Peak 3 at the end of the chromatogram results from the artifacts of the buffer.

All conditions yielded relatively high PDIs and multimodal size distributions. A minor population in the ∼40–90 nm range likely represents a monomeric or low‐order oligomeric protein, while dominant submicron to micron‐sized peaks reflect substantial heterogeneity and aggregation (Figure 4). Overall, Pc‐MBP‐Shuffle in Tris‐HCl buffer produced a more homogenous sample, although further optimization is required to obtain a more monodisperse preparation. This model is supported by the solution behavior observed in Size exclusion chromatography coupled to multi‐angle light scattering (SEC‐MALS) experiments. While monomeric Pc‐MBP exhibits features consistent with conformational flexibility, the formation of defined oligomeric species (ranging from dimers to higher‐order multimers) is accompanied by increased homogeneity and well‐defined molecular weights. Such behavior is consistent with oligomerization‐driven stabilization, where flexible regions become structurally constrained upon self‐association.

### 3.4 Oligomeric State Determined by SEC‐MALS

SEC‐MALS revealed that Pc‐MBP elutes predominantly as high molecular weight oligomers with an average molar mass of 7.49 × 10^6^ g·mol^−1^ (Figure 4). Based on the expected monomer mass of an MBP fusion protein (∼70–80 kDa), this corresponds to assemblies composed of approximately 100 subunits, demonstrating that the protein does not exist as a monomer in solution but instead forms large oligomeric particles.

The coincidence of the LS and UV signals in the combined chromatogram (Figure 4a) indicates the presence of a predominantly homogeneous protein species, consistent with a well‐defined oligomeric state in solution. However, further analysis revealed an additional population eluting at approximately 7.5 mL, designated as peak 1. Since the majority of the protein is present in peak 2, subsequent calculations of molar mass and root–mean‐square (rms) radius were performed using this fraction (Figure 4b,c). Peak 3 likely represents a chromatographic artifact arising from background noise, possibly due to minor impurities in the buffer.

As shown in Table 2, the mass distribution across the eluting peak was relatively narrow, with Mw/Mn = 1.147, indicating a uniform oligomeric population rather than uncontrolled aggregation. However, Mz/Mn = 1.552 suggests the presence of a higher‐mass tail, consistent with a minor fraction of larger species. The calculated sample mass recovery (291.41 µg) further indicates that this high‐molecular‐weight population represents a major solution species rather than a trace aggregate. SEC‐MALS also provided an average radius of gyration (Rg = 30.7 nm). Assuming near‐spherical geometry, this corresponds to an estimated particle diameter of approximately 70–80 nm, defining the size of the oligomeric assembly.

**TABLE 2:** SEC‐MALS analysis of Pc‐MBP in Tris‐HCl, 200 mM NaCl, 1 mM DTT, pH 8.0.

	Peak 1		Peak 2	
**Masses**				
Calculated Mass (μg)	413.57		291.41	
**Molar mass moments (g/mol)**				
Mn	6.621 × 10^6^	(±2.4%)	7.893 × 10^6^	(±1.6%)
Mp	7.517 × 10^6^	(±1.4%)	7.517 × 10^6^	(±1.4%)
Mv	n/a	n/a		
Mw	8.545 × 10^6^	(±1.9%)	9.050 × 10^6^	(±1.7%)
Mz	2.582 × 10^7^	(±4.3%)	1.225 × 10^7^	(±4.1%)
Mz + 1	1.943 × 10^8^	(±1.1%)	2.011 × 10^7^	(±4.4%)
M(avg)	5.898 × 10^6^	(±0.1%)	7.490 × 10^6^	(±0.1%)
**Polydispersity**				
Mw/Mn	1.291	(±3.1%)	1.147	(±2.3%)
Mz/Mn	3.899	(±4.9%)	1.552	(±4.4%)
**rms radius moments (nm)**				
Rn	26.8	(±10.3%)	26.8	(±6.8%)
Rw	29	(±7.8%)	29.1	(±6.0%)
Rz	41.8	(±3.5%)	34	(±4.7%)
R(avg)	101.9	(±0.0%)	30.7	(±0.3%)

### 3.5 Secondary Structure Measurement

**FIGURE 5:**
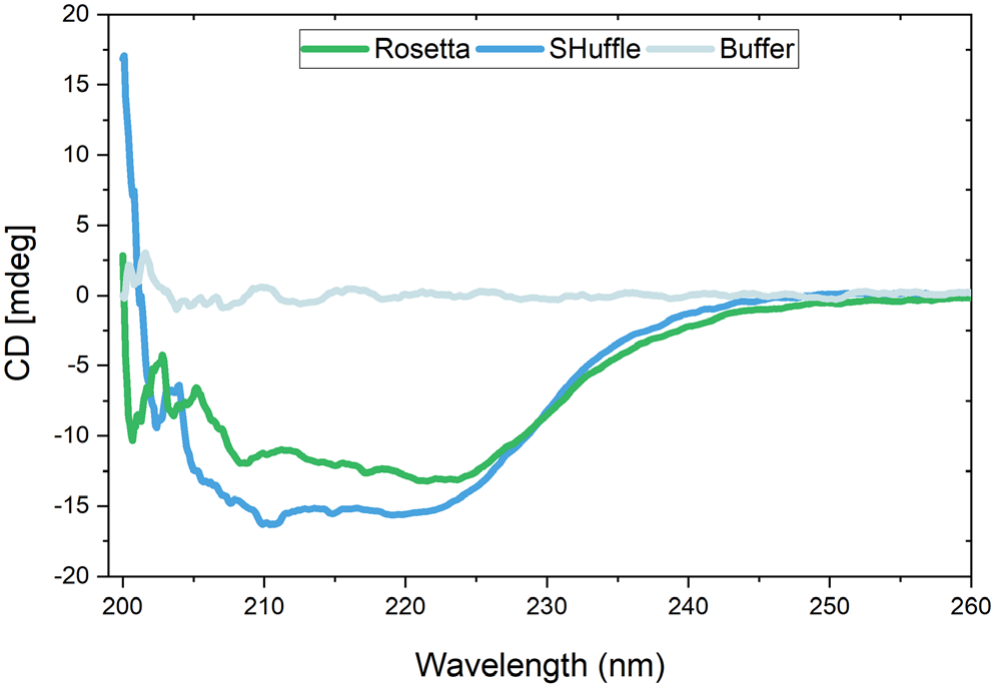
CD spectra of Pc protein. Far‐UV CD spectra of Pc protein measured under different conditions, including samples expressed in *E. coli* Rosetta cells (green), SHuffle cells (blue), and the corresponding buffer control (cyan). The spectra reveal characteristic secondary‐structure signatures of the protein, while the buffer trace confirms minimal background contribution.

To better estimate the quality of the Pc protein, two samples expressed in the bacterial strains BL21 Rosetta and BL21 SHuffle were compared. Since both samples were soluble and yielded high purification yields, the secondary‐structure content was used as a parameter to assess protein folding. For this purpose, the Far‐UV CD spectra of both samples (Pc‐MBP‐Rosetta and Pc‐MBP‐SHuffle) were recorded in their respective buffers as the background. The control buffer showed a negligible signal across the measured range, confirming that the observed spectra arise from the protein, with minimal background noise. The spectral changes correlate with changes in alpha‐helices and beta‐sheets. Both Pc‐MBP variants exhibit CD spectra characteristic of predominantly β‐sheet‐rich proteins, owing to the MBP tag, with a pronounced negative minimum around 215–220 nm and weak ellipticity near 208 nm, consistent with the expected fold of maltose‐binding protein‐derived constructs (Figure 5). Quantitative secondary‐structure analysis supports this observation (Table 3), revealing comparable β‐sheet content for Pc‐MBP‐Shuffle (36.2%) and Pc‐MBP‐Rosetta (36.4%), as well as similar turn content (∼16%). The α‐helical content is negligible in both samples (<0.5%), while nearly half of the structure is classified as “other,” likely reflecting unordered or flexible regions.

**TABLE 3:** The percentage of secondary structure profiles in Pc‐MBP derived from Shuffle and Rosetta cells.

	α‐Helix	β‐sheet	Turns	Others
Buffer	—	—		—
SHuffle	0.2	36.2	15.8	47.8
Rosetta	0.3	36.4	16.0	47.3

Despite these similarities, some subtle differences are evident in the CD spectral profiles. Pc‐MBP‐Rosetta displays a slightly more negative ellipticity in the 210–225 nm region compared to Pc‐MBP‐Shuffle. Although these differences did not translate into major changes in BeStSel‐derived secondary structure percentages, they may reflect minor variations in β‐sheet twist, strand packing, or conformational heterogeneity, features to which BeStSel is particularly sensitive. These differences can be related to strain‐dependent effects on expression, such as variations in folding efficiency, disulfide bond formation, or chaperone availability. Minor differences in intensity were also observed between the two strains at 202–205 nm, suggesting slightly higher helical content in the Pc‐MBP‐Rosetta sample.

As seen in Figure 5 and Table 3, both protein samples show characteristic negative peaks at ∼208 nm and 222 nm for the α‐helix and at ∼218 nm for the β‐sheet profile. Overall, the CD spectra are consistent with a predominantly β‐sheet–rich structure, reflecting the contribution of the MBP fusion partner. The Pc region likely remains largely disordered, as observed in AF models, and this pattern is consistent across expression hosts.

### 3.6 AF and Cryo‐EM Insights into Pc Self‐Oligomerization

MLL‐based structure predictions using AF indicate that Pc is largely intrinsically disordered, except for the N‐terminal chromodomain (Figure 1). The oligomeric models (dimer, tetramer, hexamer, and decamer) indicate a gradual increase in structural rigidity. This structural organization is well aligned with the behavior of proteins that undergo phase separation, in which IDRs mediate multivalent interactions and dynamic assembly. Consistent with experimental data, AF predictions further indicate that the C‐box region undergoes conformational tightening upon binding to C‐RING1B, suggesting that partner‐induced folding contributes to structural stabilization [14].

**FIGURE 6:**
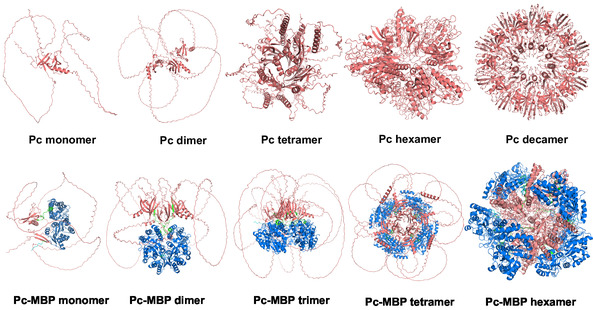
The structural organization of Pc and Pc‐MBP across monomeric and oligomeric states. The protein is mainly unstructured (upper panel); however, self‐association (e.g., tetrameric, hexameric, and decameric assemblies) and the N‐terminal MBP tag (lower panel) impart conformational rigidity. Predicted structures of MBP‐tagged Pc are shown for monomeric, dimeric, trimeric, and hexameric assemblies. Distinct domains of the fusion protein are color‐coded consistently across all models as: the His‐tag (cyan), MBP tag (blue), linked regions (green), and Pc protein (red) are indicated by distinctive colors, enabling comparison of domain organization and potential interaction interfaces across oligomeric states.

To assess the potential influence of the MBP tag on structural organization, equivalent oligomeric predictions (dimer, trimer, tetramer, and hexamer) were performed using Pc sequences lacking the MBP fusion. Structural comparisons between MBP‐tagged and untagged models were conducted to evaluate differences in domain arrangement and intermolecular interfaces. In the visualization of these models (Figure 6, lower panel), different domains and MBP‐tag are color‐coded to highlight structural organization in the higher‐order assembly of MBP‐fused proteins. These models also reveal how specific regions of Pc contribute to the spatial arrangement and folding pattern.

AF3 predictions of monomeric Pc, both in the presence and absence of the MBP tag, indicate that the protein is largely intrinsically disordered, with no well‐defined stable tertiary structure. However, upon modeling oligomeric assemblies (trimer, tetramer, hexamer, and decamer), a higher‐order architecture appears, implying a conditional folding mechanism upon oligomerization or increased local concentration. (Figure 6). These models suggest that increasing monomer number promotes intermolecular contacts with potential interfaces that stabilize local structural elements and reduce overall conformational flexibility. Notably, these AF models support a conditional folding mechanism, suggesting a model in which Pc gradually undergoes disorder‐to‐order transitions upon oligomerization, potentially contributing to its ability to form higher‐order assemblies and condensates. Such behavior is characteristic of phase‐separating systems, where local concentration, multivalency, and weak multivalent interactions drive the formation of dynamic yet functionally relevant assemblies. Given that Pc is concentrated within PcG bodies in vivo, conditional folding driven by self‐association appears biologically plausible. However, this conclusion is based on ML‐based predictions, which remain less reliable IDRs than for well‐folded proteins, necessitating experimental validation. Thus, the intrinsically disordered nature of the Pc protein, along with predicted self‐associated oligomeric structures, merits further study to clarify its role in gene silencing.

To further assess the effect of oligomerization, the sizes and radii of gyration were measured from the AF models. As shown in Table 4, the radii of gyrations are smaller than the maximum diameter (≈4x), which is consistent with the IDR nature of its long C‐terminus region. Given that Pc is enriched in the nucleus and concentrated within PcG bodies, the formation of inducible higher‐order assemblies is biologically plausible and may underlie the organizational principles of Pc‐mediated chromatin regulation [34]. However, these predicted models are generated by AF3, which has not been as accurate for IDRs as for well‐structured proteins and therefore should be considered with caution [54]. This is also evident from the very low interface predicted TM (ipTM) scores (Table 4), which is the confidence matrix for the protein subunits interface. A score of 0.3 or higher has been suggested as sufficient for estimating the oligomerization state of a protein [42]. Since most Pc models exhibit ipTM values below this threshold, the derived molecular size estimates should therefore be interpreted with caution. Despite this limitation, the models offer a valuable framework for designing and guiding subsequent experimental validation.

**TABLE 4:** Calculated molecular sizes and radii of gyration (Rg) derived from AF models. Corresponding ipTM scores are included as indicators of model confidence. Nd = not determined.

	**Predicted size** (**diameter) (Å)**	**Radius of gyration** **(Rg) (Å)**	ipTM
Pc monomer	159.4	39.5	−/nd
Pc dimer	215.0	47.5	0.15
Pc decamer	128.4	33.5	0.26
Pc undecamer	141.2	36.7	0.26
Pc‐MBP monomer	142.9	33.5	−/nd
Pc‐MBP dimer	150.0	34.8	0.2
Pc‐MBP‐tetramer	191.8	43.0	0.24
Pc‐MBP hexamer	137.1	36.7	0.29

To empirically verify the existence of such a higher‐oligomeric state, we used a cryo‐EM approach, since NMR (Figure S2) and crystallization techniques (data not shown) showed low success. Due to recent methodological advances in Cryo‐EM, structures of many small proteins of ≈50 KDa have been solved [55]. Given that the Pc protein has a molecular weight of ∼44 kDa as a monomer and ∼85 kDa when fused to an MBP tag, cryo‐EM represents a suitable approach for its structural characterization.

**FIGURE 7:**
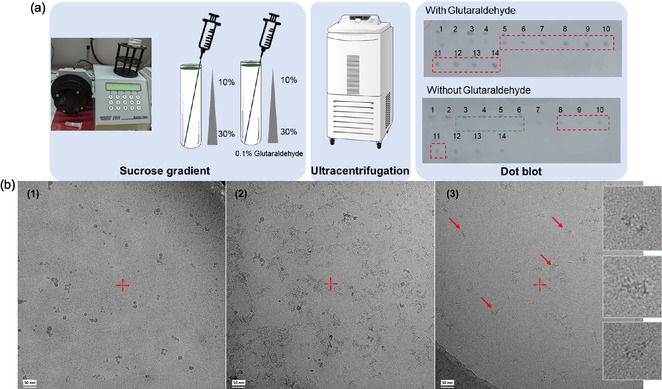
Upper panel: sample preparation via GraFix before grid preparation. The samples from ultracentrifugation fractions were analyzed via Dot blot for size estimation of fixed and unfixed protein samples. The green box in the lower Dot blot shows the disappearance of smaller protein fractions. Lower Panel: CryoEM images of Pc‐MBP acquired (1) after concentrating the purified protein, (2) after fixing the concentrated sample with 0.1% Glutaraldehyde, and (3) after GraFix treatment. The representative images of the GraFix sample show the presence of particles with a homogeneous size distribution. The scale bar in all images is equal to 50 nm.

Initial Cryo‐EM analysis of Pc‐MBP protein revealed pronounced heterogeneity in both oligomeric state and particle conformation, consistent with a dynamic and weakly stabilized assembly (Figure 7a). The observed oligomeric states were highly sensitive to grid preparation, indicating that intermolecular interactions governing Pc self‐association are readily perturbed during vitrification. Such sensitivity is characteristic of assemblies stabilized by multivalent, low‐affinity interactions, as commonly observed in proteins involved in biomolecular condensates and phase‐separated nuclear compartments.

Further, the protein was cross‐linked with Glutaraldehyde using the GraFix method. This approach partially mitigated the sample heterogeneity (Figure 7b) and improved the size distribution while preserving the weak intermolecular interactions. The GraFix‐stabilized samples yielded particles with sizes more consistent with those predicted by AF‐based structural models (Figure 6). This observation suggests that mild crosslinking can preserve higher‐order Pc assemblies that may otherwise dissociate during grid preparation. However, even under these conditions, substantial conformational and oligomeric variability persisted, underscoring the intrinsic plasticity of Pc self‐association. Further optimization, including screening of buffer conditions, stabilizing additives, or alternative crosslinkers, will likely be required to obtain a more homogeneous sample suitable for high‐resolution structure determination. In addition, the incorporation of DNA oligonucleotides or known Pc‐binding partners may promote assembly stabilization by mimicking physiological interactions within nuclear Pc bodies.

In summary, cryo‐EM analysis of purified Pc in the absence of crosslinking did not reveal the large oligomeric particles (∼7 MDa) detected by DLS and SEC‐MALS, likely due to partial dissociation during grid preparation. In contrast, GraFix‐treated samples preserved larger assemblies with dimensions more consistent with AF3‐predicted oligomeric states (Figure 6, Table 4). However, substantial variability in particle size and shape is still a limitation in the high‐resolution structural reconstruction, indicating significant conformational and compositional heterogeneity.

Previous studies on PRCs have also demonstrated highly dynamic and transient interactions or Pc proteins with chromatin rather than stable occupancy at target sites [56]. Furthermore, both in vitro and cellular studies have shown that PRC1 member proteins can form modular and condensate‐like assemblies whose properties strongly depend on subunit composition and chromatin context [28, 39]. Together, these findings support the conformational plasticity and buffer‐dependent oligomerization behavior observed for Pc in this study, suggesting that Pc and related PRC1 proteins may rely on transient multivalent interactions rather than rigid, well‐defined assemblies for their biological function.

Despite the limitations associated with sample heterogeneity, our results provide initial experimental evidence that Pc can form self‐associated assemblies and potentially adopt multiple oligomeric states in solution. Future studies aimed at improving sample homogeneity through optimization of buffer conditions, stabilizing additives, crosslinking strategies, or incorporation of interaction partners (PRC1) will be important for resolving structurally defined Pc assemblies at higher resolution.

## 4 Conclusion

In this study, we establish a comprehensive biochemical and biophysical framework for investigating the Pc protein, a key but structurally challenging component of PRC1. By combining solubility‐enhancing fusion strategies with systematic optimization of expression, purification, and buffer conditions, we successfully generated stable Pc constructs in quantities suitable for advanced biophysical and solution‐based analyses. Our results confirm that Pc is largely intrinsically disordered outside its chromodomain, yet potentially capable of forming higher‐order oligomeric assemblies that exhibit improved structural organization when combined with a solubility‐enhancing fusion partner. It is important to mention that almost all biochemical and structural characterizations in this study were performed on the MBP‐tagged form of Pc, as removal of the tag resulted in complete protein precipitation, thereby limiting analysis of the untagged protein. Future work will benefit from exploring alternative solubilization or refolding strategies to enable tag‐free studies.

Thermal stability assays, nanoDSF, DLS, and SEC‐MALS collectively reveal that Pc self‐association is strongly influenced by buffer composition, temperature, and storage conditions, with HEPES and Tris‐based buffers providing optimal stabilization. Cryo‐EM analysis further highlights the pronounced conformational and oligomeric heterogeneity of Pc, a feature consistent with proteins involved in biomolecular condensates and phase‐separated nuclear compartments. Mild chemical stabilization using the GraFix approach preserves larger Pc assemblies and enables visualization of intact oligomeric particles, although substantial heterogeneity remains a limiting factor for high‐resolution structure determination. Importantly, AF‐based predictions suggest a conditional folding mechanism in which Pc adopts more ordered architectures upon self‐association, providing a conceptual link between intrinsic disorder, oligomerization, and functional assembly within Polycomb bodies. While these models are fairly predictive and require further experimental validation, they offer a probable structural basis for Pc‐mediated chromatin organization and gene silencing.

Overall, this work lays the groundwork for future integrative structural studies of Pc and related Polycomb proteins. By addressing key technical challenges associated with intrinsically disordered and self‐associating proteins, our findings contribute to a deeper understanding of how structural plasticity underpins epigenetic regulation and nuclear organization.

## Funding

This study was supported by Niedersächsisches Ministerium für Wissenschaft und Kultur (12.5‐76251‐17‐9/20); AUFF (AUFF‐T‐2018‐7‐11).

## Conflicts of Interest

The authors declare no conflicts of interest.

## Supporting information


The additional data on MBP tag removal, NMR spectra, DLS, and Cryo‐EM are provided in the supporting information.

## Data Availability

The data that support the findings of this study are available from the corresponding author upon reasonable request.
