# Supplementary material for: Comprehensive Biophysical Profiling Evidences Self‐Oligomerization of Bacterially Expressed Pc Protein
Source: Chembiochem. 2026 Jun 13;27(11):e70405. doi: 10.1002/cbic.70405 (PMC13264468; doi:10.1002/cbic.70405)
Supplement: Supplementary file 1 — The additional data on MBP tag removal, NMR spectra, DLS, and Cryo‐EM are provided in the supporting information. [file CBIC-27-e70405-s001.pdf]

Supplementary Information

Comprehensive Biophysical Profiling Evidences Self-Oligomerization of Bacterially Expressed Pc Protein

Maria Zahid[a]*, Sabin Prajapati[b], Ghazaleh Alamdari[a], Kai Tittmann[b], and Selin Kara[a,c]*

In memory of Prof. Iván Lavandera García, our beloved and dear colleague and friend.

[a] Dr. Maria Zahid, Ghazaleh Alamdari, Prof. Dr.-Ing. habil. Selin Kara
Institute of Technical Chemistry
Leibniz University Hannover, Callinstr. 5, 30167 Hannover, Germany
E-mail: zahid@iftc.uni-hannover.de, selin.kara@iftc.uni-hannover.de

[b] Dr. Sabin Prajapati, Prof. Dr. Kai Tittmann
Department of Molecular Enzymology
Göttingen Centre for Molecular Biosciences (GZMB) and Albrecht-von-Haller-Institute
Georg-August-University Göttingen, Germany

[c] Prof. Dr.-Ing. habil. Selin Kara
Biocatalysis and Bioprocessing Group, Department of Biological and Chemical Engineering
Aarhus University
Gustav Wieds Vej 10, 8000 Aarhus, Denmark
E-mail: selin.kara@bce.au.dk

WILEY-VCH

Table of Contents

Methods 3
Sequence analysis and physicochemical properties 3
Disorder prediction 3
Structure prediction 3
Electrostatic surface profile calculation 3
Construct design and gene synthesis 3
Bacterial expression and purification 3
SDS-PAGE and Western Blot (WB) 4
Tag removal via TEV protease 4
Thermofluor (TF) assay 4
NMR spectroscopy 4
Results and Discussion 5
Challenges with the MBP tag removal 5
NMR analysis 6
Buffer optimization using TF assay 6
Thermal stability via Nano-DSF measurements 8
Protein molecular weight and oligomerization 9
References 10

WILEY-VCH

Methods

Sequence analysis and physicochemical properties

The amino acid sequence of Pc-MBP was analyzed using the ProtParam tool (https://web.expasy.org/protparam/) to calculate basic physicochemical properties, including molecular weight, theoretical isoelectric point (pI), amino acid composition, extinction coefficient, instability index, aliphatic index, and grand average of hydropathicity (GRAVY) [1]. These parameters were used to assess the overall biochemical characteristics of the protein.

Disorder prediction

Intrinsic disorder in the Pc protein was predicted using AIUPred**,** which combines energy estimation with a transformer-based deep learning model [2]. The full-length Pc protein sequence was analyzed using default parameters, and per-residue disorder scores were calculated. The output was visualized as a plot of disorder propensity values ranging from 0 to 1, with higher scores indicating an increased likelihood of intrinsic disorder.

Structure prediction

Protein structure predictions were generated using AlphaFold 3 [3]. The full-length Pc-MBP amino acid sequence was used as input for both prediction pipelines with default parameters. Predicted models were evaluated based on confidence metrics provided by each method, including per-residue confidence scores. Structural models were visualized and compared to assess overall fold consistency and the presence of ordered versus disordered regions.

Electrostatic surface profile calculation

Electrostatic surface potentials of the Pc protein with and without the N-terminal MBP tag were calculated using the APBS electrostatics plugin implemented in PyMOL [4]. Before calculation, structures were prepared by assigning appropriate protonation states and adding missing hydrogens. Electrostatic potentials were computed by solving the Poisson–Boltzmann equation under default parameters. Electrostatic potentials were visualized on a normalized scale (−5 to +5 kT/e) to allow direct comparison between constructs.

Construct design and gene synthesis

For biophysical and structural studies, we need protein in mg/mL amounts for which *E. col*i is often a good choice. However, recombinant expression of eukaryotic proteins in *E. coli* is usually hindered by the formation of insoluble inclusion bodies. To address this challenge, a solubility-enhancing fusion tags - maltose-binding protein (MBP) was evaluated for its ability to improve the solubility and expression of the Pc protein from *Drosophila melanogaster*. To dissect the roles of different domains, five constructs were designed (as shown in Figure 1). In these constructs, full-length (FL) and truncated forms of the Pc protein, covering the N- and C-terminal domains, were designed with MBP as a fusion partner to enhance solubility. A His-tag before the MBP-tag was also added to facilitate purification and identification of the expressed proteins.

Bacterial expression and purification

The MBP-tagged Pc protein was recombinantly expressed in two *E. coli* strains, Rosetta 2 and SHuffle T7, in Terrific Broth (TB) medium. The expression temperature before and after induction was 37°C and 18°C, respectively, at 200 rpm. The induction phase was initiated at an OD_{600} of 0.5 by adding 0.25 mM IPTG to a final concentration. Samples were collected at various time points during cultivation to monitor protein expression and solubility. SDS–PAGE analysis revealed that induction with 0.25 mM IPTG resulted in a higher proportion of soluble Pc-MBP compared to induction with 0.5 mM IPTG (data not shown), indicating that lower induction strength favors proper folding and solubility of the fusion protein.

Pc-MBP expressed in Rosetta 2 and SHuffle T7 was subsequently purified using fast protein liquid chromatography (FPLC) through sequential affinity chromatography (HisTrap and MBPTrap) followed by size-exclusion chromatography (SEC). The cell pellets from 1 L cultures were resuspended in 30 mL lysis buffer (50 mM Tris-HCl, pH 7.5, 200 mM NaCl, 1 mM EDTA, 1 mM DTT, 1 mM Triton-X, 1 mM PMSF, 1 mg/mL lysozyme, and 25-50 µg/mL DNase). Cells were lysed using a Sonicator (TS102) or a Microfluidizer for small- or large-scale samples.

WILEY-VCH

For small-scale samples, the cell pellet was resuspended in 100 µL of lysis buffer and sonicated with 10-second on/30-second off pulses at 50% amplitude. Typically, 3–4 sonication cycles were sufficient for time-point samples; for smaller samples, 2-second on/8-second off pulses were used. For biomass up to 5 g, 6-8 cycles were required to achieve efficient lysis.

SDS-PAGE and Western Blot (WB)

For the qualitative and quantitative analyses of proteins, the lysate samples were centrifuged after sonication at maximum speed (17,000 × g, 4°C) for 10 minutes. The supernatant, representing the soluble fraction, was transferred to a fresh microcentrifuge tube, and 20 µL of 6× Laemmli buffer was added. The pellet, corresponding to the insoluble fraction, was resuspended in 100 µL of 1× Laemmli buffer. All samples were heated at 98°C for 8 minutes, briefly centrifuged, and subsequently analyzed by SDS–PAGE and Western blotting. The soluble and insoluble fractions of cell lysates were separately mixed with Laemmli dye (375 mM Tris-HCl, pH 6.8, 9% SDS, 50% glycerol, and 0.06% bromophenol blue), heated to 95°C for 8 minutes, and centrifuged. 10 µL of each sample was loaded onto a 10% SDS-PAGE. The gel was run at 200 V for 45 minutes, and the proteins were visualized overnight with colloidal Coomassie Dye.

For Western blotting, the samples were prepared as for SDS-PAGE. The gel was transferred to a PVDF membrane using a semi-dry apparatus (TransBlot SD, BioRad). The proteins were blotted with HRP-conjugated anti-His antibody (MA1-21315-HRP, Invitrogen) at room temperature with gentle shaking. The detection was done by using chromogenic signal via 1-step TMB solution (ThermoScientific). The blot was washed in MilliQ water and documented via gel scanning software.

Tag removal via TEV protease

To study the effect of the MBP tag on protein solubility, ~300 mg of the purified Pc-MBP protein was treated with TEV protease (~2.2 ug/uL), in cleavage buffer (50 mM Tris-HCl pH 8.0, 150 mM NaCl, 0.5 mM EDTA, 1 mM DTT). The reaction was carried out in a dialysis membrane (MWCO 10 KDa) at 4 °C overnight (or at 16 °C for ~4h) with gentle mixing in the dialysis bath. Tag cleavage efficiency was assessed by SDS-PAGE.

Following digestion, the MBP tag and TEV protease were removed by a reverse-affinity chromatography step (MBPtrap column, Cytiva), and the cleaved Pc protein was collected in the flow-through (FT). The eluted protein was subsequently concentrated using Amicon centrifugal filter units (Millipore) to the desired concentration. The final protein samples were centrifuged at 13.3K rpm, 10 min, 4°C, to remove the aggregates.

Thermofluor (TF) assay

The buffer optimization for truncated constructs was performed using a thermal shift assay in a microtiter plate (MTP) format [5]. Briefly, 21 µL of each buffer condition was dispensed into individual wells, along with water as a blank control. 2 µL of purified samples (20-100 µM) was added to each well, together with 2 µL of 62.5X SYPRO Orange dye (Invitrogen). The plates were quickly spun to collect the reaction mixture, and sealed with optical adhesive films. The thermal denaturation analysis was performed on an RT-PCR machine using a temperature gradient of 1 °C/min from 5 to 95 °C. The maximum excitation and emission wavelengths of SYPRO Orange were used at 470 and 569 nm, respectively.

NMR spectroscopy

NMR spectra of isotopically labeled Pc protein were acquired using transverse relaxation-optimized spectroscopy (TROSY) experiments as previously reported protocol [6]. Measurements were performed at 298 K on a high-field spectrometer equipped with a cryogenic probe. Samples were prepared in NMR buffer (50 mM Tris-HCl pH 8.0, 150 mM NaCl, 1 mM DTT, 5-10% D_2O) at ~200 mg/mL protein concentration. Two-dimensional [^1H,^{15}N]-TROSY spectra were recorded to assess protein folding and backbone amide signal dispersion. Data were processed and analyzed using TopSpin software (Bruker BioSpin GmbH).

WILEY-VCH

Results and Discussion

Challenges with the MBP tag removal

In addition to the full-length protein, several truncated constructs were generated to dissect the roles of individual domains. For all constructs, attempts to remove the MBP tag using TEV protease consistently resulted in rapid aggregation of the Pc proteins. Multiple cleavage conditions were tested, including incubation at 4 °C versus 16 °C, short (4 h) versus overnight reactions, and both in-solution (dialysis tubing) and on-column cleavage approaches; however, all conditions led to substantial protein precipitation (Figure S1c–d). Although a small fraction of soluble Pc could be recovered following cleavage (Figure S1-e), the overall yield was extremely low, impeding further biochemical and structural analyses.

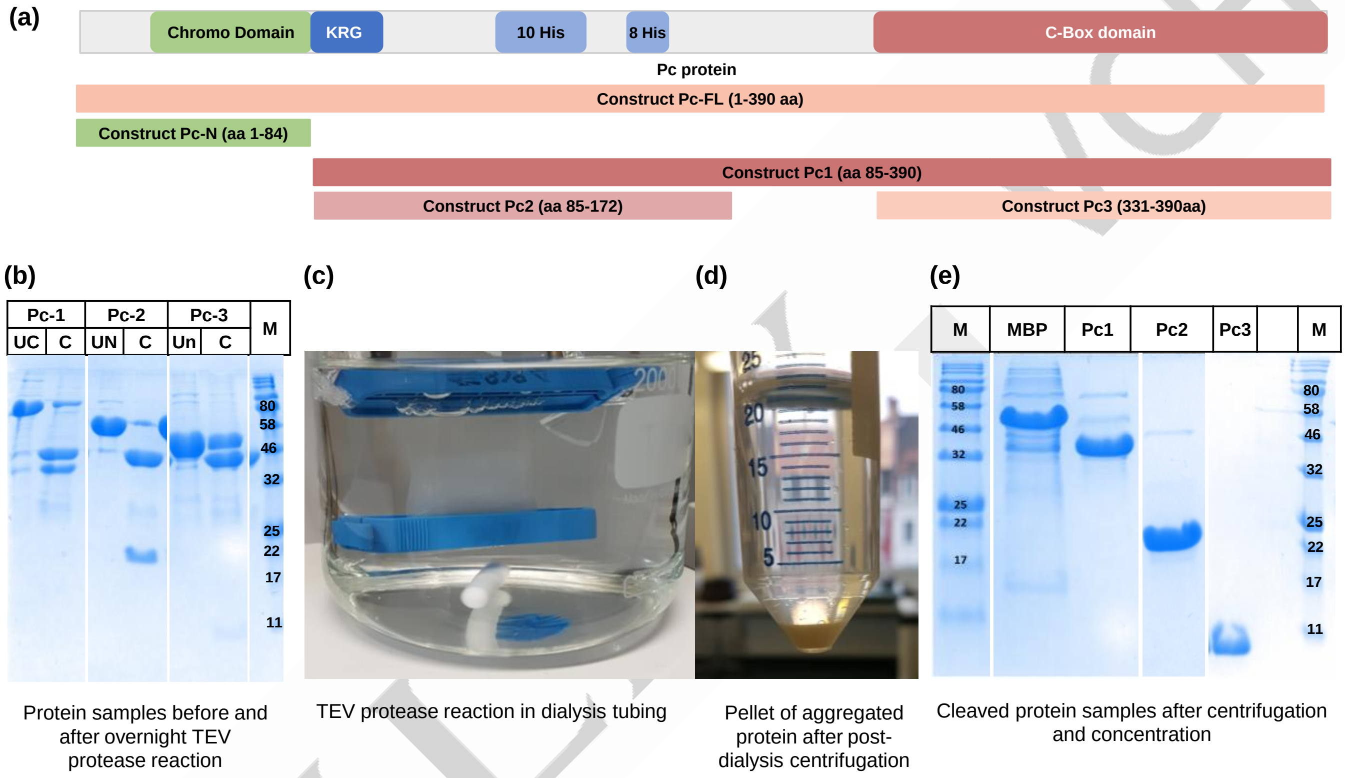

Figure S1. TEV-mediated cleavage of Pc-MBP and aggregation upon tag removal. (a) Design of different Pc constructs used for MBP-fused expression and purification. (b) SDS-PAGE analysis of Pc-MBP before cleavage (UN: uncut) and after TEV protease treatment (c: cut) demonstrates efficient cleavage of the fusion protein. (c–d) Following tag removal and dialysis, the cleaved Pc protein formed visible precipitates, as shown by the centrifuged sample, indicating aggregation upon loss of the MBP tag. (e) SDS-PAGE analysis of the proteins (Pc1, Pc2 & Pc3) recovered in the soluble fraction after concentration using an Amicon centrifugal filter (10 kDa MWCO).

Upon removal of the MBP tag, the protein precipitated and could not be efficiently resolubilized under denaturing conditions using urea or guanidine hydrochloride (data not shown). Attempts to recover the protein using simple refolding strategies were performed, but did not result in efficient refolding. These conditions were not explored exhaustively and may warrant further optimization in future studies. This behavior indicates that Pc is intrinsically prone to aggregation when expressed in isolation, which was also observed in three different truncated constructs without the MBP-tag (data not shown). Altogether, this suggests that the MBP tag plays a critical role in maintaining solubility, likely by stabilizing partially folded or disordered states. It is consistent with well-documented ability of the MBP to markedly enhance the solubility, and in many cases, promoting proper folding of aggregation-prone fusion partners. The pronounced dependence on MBP for solubility observed here may therefore reflect an intrinsic tendency of Pc to aggregate during heterologous expression in *E. coli*, potentially occurring prior to the acquisition of its native conformation [7]. Due to this stark effect of tag removal, all further analyses (except the NMR measurements or mentioned otherwise) were performed with the Pc-MBP fusion protein.

NMR analysis

Deuterium-labeled Pc proteins were purified as described in the main text, treated with TEV protease, concentrated, and centrifuged at 13,300 rpm at 4 °C to remove any aggregates. TROSY spectra of the labelled proteins were acquired following the previously reported protocol. For all constructs (PcC1, PcC2, and PcC3), the spectra displayed broad, merged signals lacking well-resolved peaks, precluding assignment to the amino acid backbone (Figure S2).

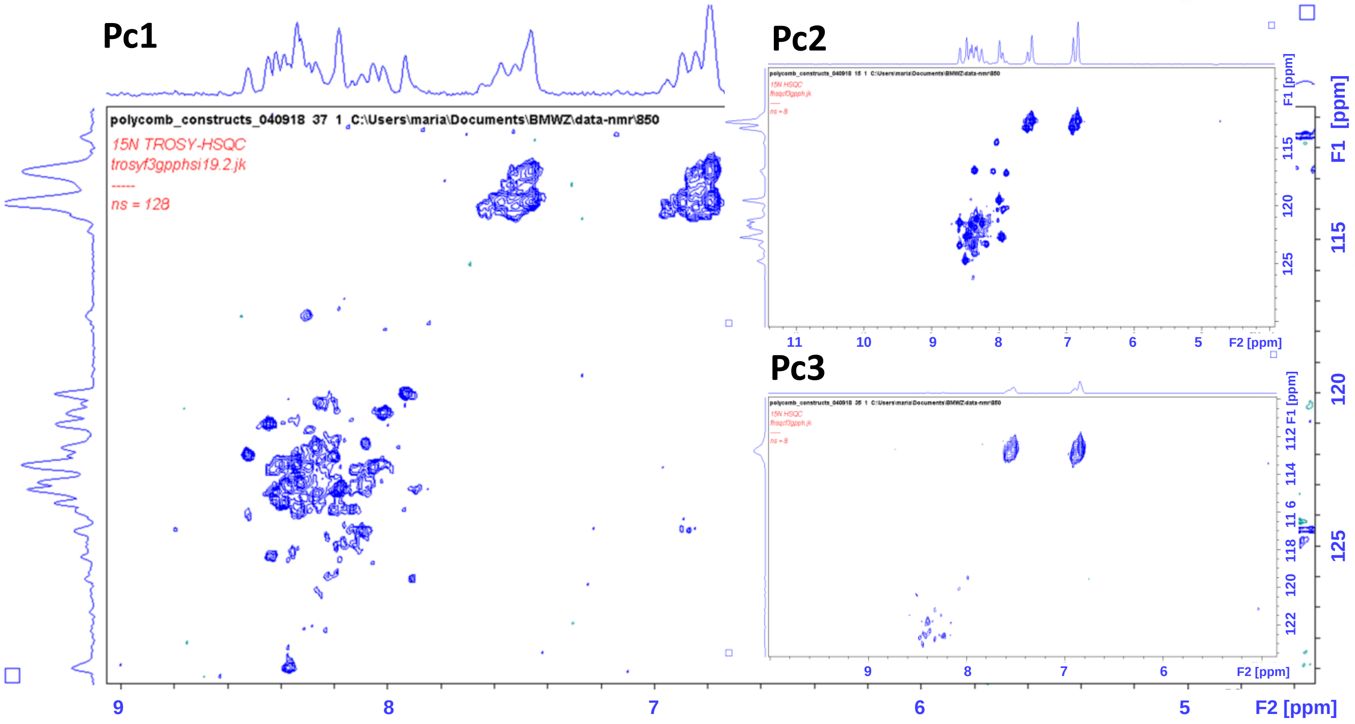

Figure S2. 15N TROESY spectra of the 15N-labelled Pc proteins. The upper panel shows the individual spectra of three constructs, Pc1, Pc2 and Pc3. The lower panel shows an inset of the signals observed in Pc1. The signals lack sharp peaks and therefore cannot be assigned to the residue backbone.

Control experiments using unlabeled protein samples yielded comparable spectral features (data not shown), indicating that the observed signal broadening is not due to labeling effects. Instead, these results suggest that the large effective molecular size arising from self-association renders Pc unsuitable for high-resolution NMR analysis.

Buffer optimization using TF assay

The previously established TF assay was used to screen various buffer conditions (Figure S3a) for the purified MBP-tagged Pc protein. Based on the highest scores, Tris-HCl, HEPES, and MOPS buffers, among others, were selected for thermal stability studies.

(a)

	1	2	3	4	5	6	7	8	9	10	11	12
A	water	50mM Na*Acetate<br>pH 5.0	50mM Ammonium Acetate<br>pH 5.0	50mM Na*Citrate<br>pH 5.5	50mM Na*phosphate<br>(monobasic)<br>pH 6.0	50mM K*Phosphate<br>(monobasic)<br>pH 6.0	50mM MES<br>pH 6.0	50mM MES / 50 mM Bis-Tris<br>pH 6.0	50mM Bis-Tris / 50mM PIPES<br>pH 6.5	50mM Bis-Tris<br>pH 6.5	50mM Na*Phosphate<br>(monobasic)<br>pH 7.0	50mM K*Phosphate<br>(monobasic)<br>pH 7.0
B	50mM HEPES<br>pH 7.0	50mM MOPS / 50 mM Bis-Tris propane<br>pH 7.0	50mM MOPS<br>pH 7.0	50mM Tris-HCl<br>pH 7.5	50mM Na*Phosphate<br>(monobasic)<br>pH 7.5	50mM K*Phosphate<br>(monobasic)<br>pH 7.5	50mM Tris-HCl<br>pH 8.0	50mM Bicine<br>pH 8.0	50mM Na*Acetate<br>pH 5.0<br>100mM NaCl	50mM Ammonium Acetate<br>pH 5.0<br>100mM NaCl	50mM Na*Citrate<br>pH 5.5<br>100mM NaCl	50mM Na*phosphate<br>(monobasic) pH 6.0<br>100mM NaCl
C	50mM K*Phosphate<br>(monobasic) pH 6.0<br>100mM NaCl	50mM MES<br>pH 6.0<br>100mM NaCl	50mM MES / 50 mM Bis-Tris<br>pH 6.0<br>100mM NaCl	50mM Bis-Tris / 50mM PIPES<br>pH 6.5<br>100mM NaCl	50mM Bis-Tris<br>pH 6.5<br>100mM NaCl	50mM Na*Phosphate<br>(monobasic) pH 7.0<br>100mM NaCl	50mM K*Phosphate<br>(monobasic) pH 7.0<br>100mM NaCl	50mM HEPES<br>pH 7.0<br>100mM NaCl	50mM MOPS / 50 mM Bis-Tris propane pH 7.0<br>100mM NaCl	50mM MOPS<br>pH 7.0<br>100mM NaCl	50mM Tris-HCl<br>pH 7.5<br>100mM NaCl	50mM Na*Phosphate<br>(monobasic) pH 7.5<br>100mM NaCl
D	50mM K*Phosphate<br>(monobasic) pH 7.5<br>100mM NaCl	50mM Tris-HCl<br>pH 8.0<br>100mM NaCl	50mM Bicine<br>pH 8.0<br>100mM NaCl	50mM Na*Citrate<br>pH 5.5<br>20mM NaCl	50mM Na*Citrate<br>pH 5.5<br>50mM NaCl	50mM Na*Citrate<br>pH 5.5<br>200mM NaCl	50mM Na*Citrate<br>pH 5.5<br>500mM NaCl	50mM MES / 50 mM Bis-Tris<br>pH 6.0<br>20mM NaCl	50mM MES / 50 mM Bis-Tris<br>pH 6.0<br>50mM NaCl	50mM MES / 50 mM Bis-Tris<br>pH 6.0<br>200mM NaCl	50mM MES / 50 mM Bis-Tris<br>pH 6.0<br>500mM NaCl	50mM Bis-Tris<br>pH 6.5<br>20mM NaCl
E	50mM Bis-Tris<br>pH 6.5<br>50mM NaCl	50mM Bis-Tris<br>pH 6.5<br>200mM NaCl	50mM Bis-Tris<br>pH 6.5<br>500mM NaCl	50mM HEPES<br>pH 7.0<br>20mM NaCl	50mM HEPES<br>pH 7.0<br>50mM NaCl	50mM HEPES<br>pH 7.0<br>200mM NaCl	50mM HEPES<br>pH 7.0<br>500mM NaCl	50mM Na*Phosphate<br>(monobasic) pH 7.0<br>20mM NaCl	50mM Na*Phosphate<br>(monobasic) pH 7.0<br>50mM NaCl	50mM Na*Phosphate<br>(monobasic) pH 7.0<br>200mM NaCl	50mM Na*Phosphate<br>(monobasic) pH 7.0<br>500mM NaCl	50mM Na*Citrate<br>pH 5.5<br>20mM KCl
F	50mM Na*Citrate<br>pH 5.5<br>50mM KCl	50mM Na*Citrate<br>pH 5.5<br>200mM KCl	50mM Na*Citrate<br>pH 5.5<br>500mM KCl	50mM MES / 50 mM Bis-Tris<br>pH 6.0<br>20mM KCl	50mM MES / 50 mM Bis-Tris<br>pH 6.0<br>50mM KCl	50mM MES / 50 mM Bis-Tris<br>pH 6.0<br>200mM KCl	50mM MES / 50 mM Bis-Tris<br>pH 6.0<br>500mM KCl	50mM Bis-Tris<br>pH 6.5<br>20mM KCl	50mM Bis-Tris<br>pH 6.5<br>50mM KCl	50mM Bis-Tris<br>pH 6.5<br>200mM KCl	50mM Bis-Tris<br>pH 6.5<br>500mM KCl	50mM HEPES<br>pH 7.0<br>20mM KCl
G	50mM HEPES<br>pH 7.0<br>50mM KCl	50mM HEPES<br>pH 7.0<br>200mM KCl	50mM HEPES<br>pH 7.0<br>500mM KCl	50mM Na*Phosphate<br>(monobasic) pH 7.0<br>20mM KCl	50mM Na*Phosphate<br>(monobasic) pH 7.0<br>50mM KCl	50mM Na*Phosphate<br>(monobasic) pH 7.0<br>200mM KCl	50mM Na*Phosphate<br>(monobasic) pH 7.0<br>500mM KCl	20mM Na*Phosphate<br>(monobasic)<br>pH 7.0	100mM Na*Phosphate<br>(monobasic)<br>pH 7.0	200mM Na*Phosphate<br>(monobasic)<br>pH 7.0	5mM DTT<br>50mM Na*Phosphate pH 7.0<br>50mM NaCl	2mM TCEP<br>50mM Na*Phosphate pH 7.0<br>50mM NaCl
H	10mM Mg*Chloride<br>50mM Na*Phosphate pH 7.0<br>50mM NaCl	10mM Ca*Chloride<br>50mM Na*Phosphate pH 7.0<br>50mM NaCl	10mM Na*Sulfate<br>50mM Na*Phosphate pH 7.0<br>50mM NaCl	20mM Proline<br>50mM Na*Phosphate pH 7.0<br>50mM NaCl	50mM Arginine<br>50mM Na*Phosphate pH 7.0<br>50mM NaCl	50mM Glutamate<br>50mM Na*Phosphate pH 7.0<br>50mM NaCl	50mM Arg + 50mM Glu<br>50mM Na*Phosphate pH 7.0<br>50mM NaCl	20mM Glycine<br>50mM Na*Phosphate pH 7.0<br>50mM NaCl	50mM D-glucose<br>50mM Na*Phosphate pH 7.0<br>50mM NaCl	2mM CHAPS<br>50mM Na*Phosphate pH 7.0<br>50mM NaCl	1mM Octyl glucoside<br>50mM Na*Phosphate pH 7.0<br>50mM NaCl	5mM EDTA<br>50mM Na*Phosphate pH 7.0<br>50mM NaCl

(b)

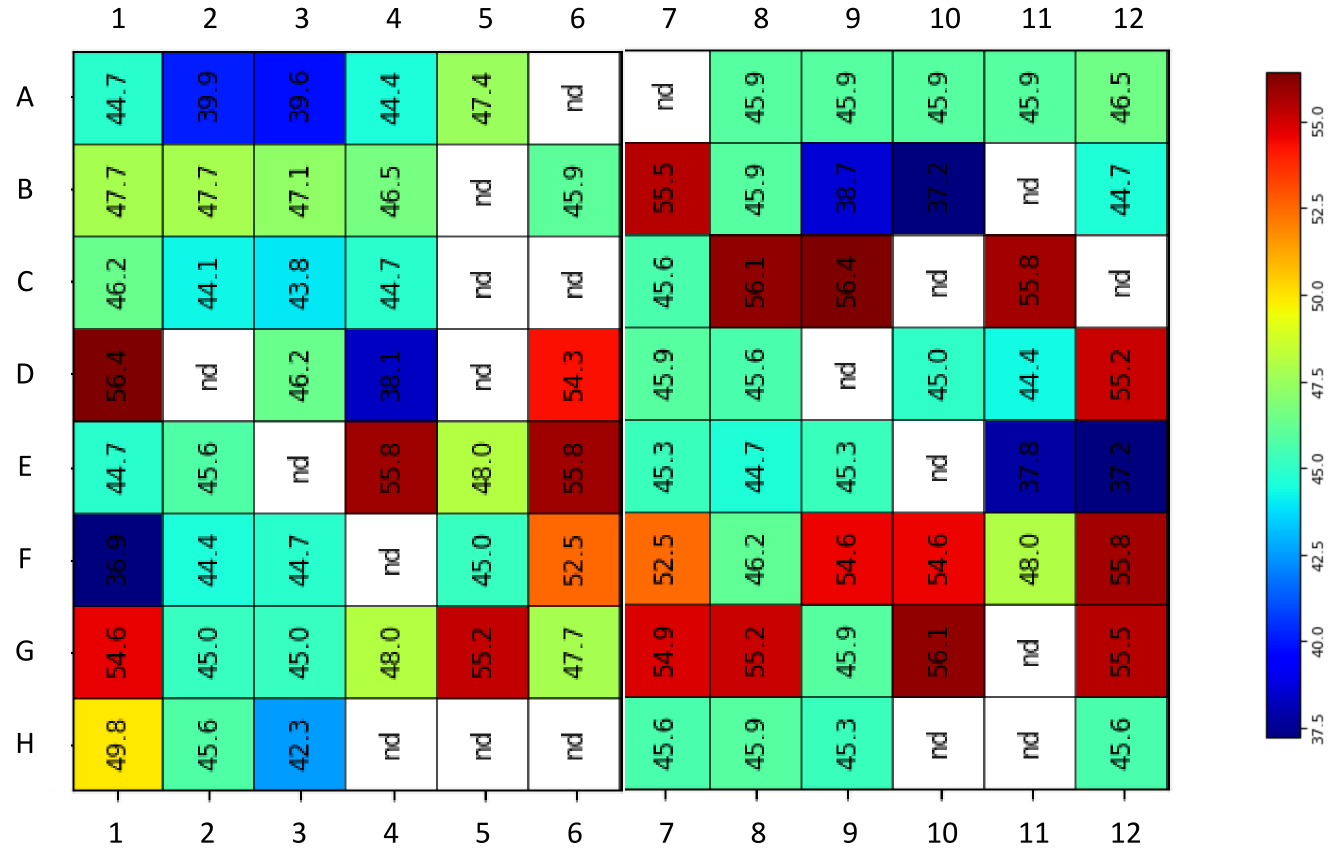

Figure S3. Thermofluor (TF) assay of Pc-MBP protein. The purified protein was incubated with SYPRO Orange dye, and the inflection points were measured in an RT-PCR machine. The composition of the buffer screen is shown in the upper panel (S3-a). The Tms in these buffer conditions are shown in the form of a color matrix in the lower panel (S3-b). The scale bar on the right side shows the range of Tm values, from low (blue) to high (red). Nd: not determined

Thermal stability via Nano-DSF measurements

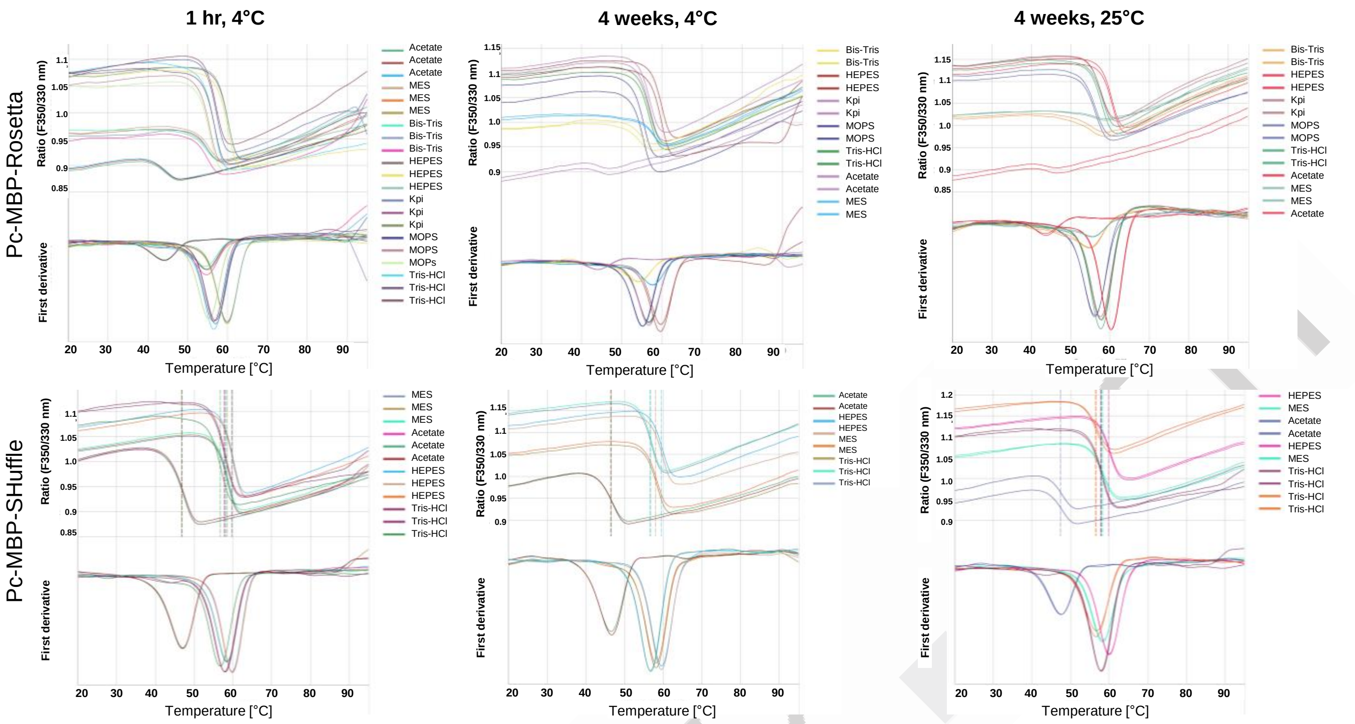

Figure S4. Thermal stability of Pc-MBP expressed in *E. coli* Rosetta (upper panel) and *E. coli* Shuffle (lower panel) strains in different buffer systems using NanoDSF. Samples were prepared in the indicated buffer systems (HEPES, Tris-HCl, KPi, MOPS, MES, Bis-Tris, and Acetate). The melting curves and corresponding first derivatives of the Pc protein were measured after 1 h at 4 °C, 4 weeks at 4 °C, and 4 weeks at 25 °C. Fluorescence intensity ratios (F350/F330) are plotted as a function of temperature, and melting transitions are highlighted by minima in the first-derivative curves, corresponding to apparent melting temperatures (Tm). These spectra were visualized via PR Stability Analysis software (v1.1, NanoTemper Technologies).

The data shown in Figure S4 illustrate buffer- and storage-dependent effects on the thermal stability of the Pc protein. Notably, the Pc protein exhibits a blue shift in the apparent Tm, as observed in the first-derivative profiles, which can be attributed to the predominantly hydrophilic nature of the protein.

WILEY

Protein molecular weight and oligomerization

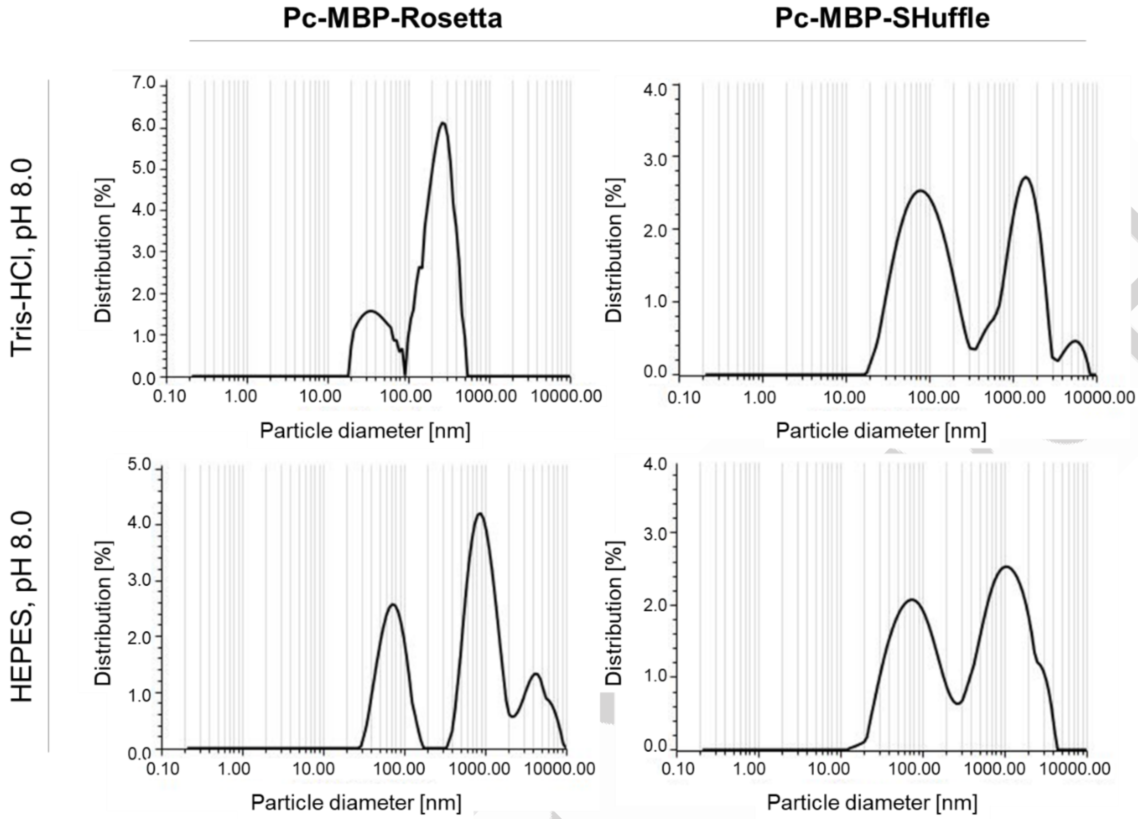

Figure S5. Dynamic light scattering (DLS) to analyze the size distribution of protein samples (Pc-MBP-SHuffle T7 and Pc-MBP-Rosetta 2) in the buffers Tris-HCl and HEPES.

References

[1] M. R. Wilkins, E. Gasteiger, A. Bairoch, J. C. Sanchez, K. L. Williams, R. D. Appel and D. F. Hochstrasser, Protein identification and analysis tools in the ExPASy server, *Methods in molecular biology*, **1999**, 112, 10.1385/1-59259-584-7:531.

[2] G. Erdos and Z. Dosztanyi, AIUPred: combining energy estimation with deep learning for the enhanced prediction of protein disorder, *Nucleic acids research*, **2024**, 52, 10.1093/nar/gkae385.

[3] J. Abramson, J. Adler, J. Dunger, R. Evans, T. Green, A. Pritzel, O. Ronneberger, L. Willmore, A. J. Ballard, J. Bambrick, S. W. Bodenstein, D. A. Evans, C. C. Hung, M. O'Neill, D. Reiman, K. Tunyasuvunakool, Z. Wu, A. Zemgulyte, E. Arvaniti, C. Beattie, O. Bertolli, A. Bridgland, A. Cherepanov, M. Congreve, A. I. Cowen-Rivers, A. Cowie, M. Figurnov, F. B. Fuchs, H. Gladman, R. Jain, Y. A. Khan, C. M. R. Low, K. Perlin, A. Potapenko, P. Savy, S. Singh, A. Stecula, A. Thillaisundaram, C. Tong, S. Yakneen, E. D. Zhong, M. Zielinski, A. Zidek, V. Bapst, P. Kohli, M. Jaderberg, D. Hassabis and J. M. Jumper, Accurate structure prediction of biomolecular interactions with AlphaFold 3, *Nature*, **2024**, 630, 10.1038/s41586-024-07487-w.

[4] E. Jurrus, D. Engel, K. Star, K. Monson, J. Brandi, L. E. Felberg, D. H. Brookes, L. Wilson, J. Chen, K. Liles, M. Chun, P. Li, D. W. Gohara, T. Dolinsky, R. Konecny, D. R. Koes, J. E. Nielsen, T. Head-Gordon, W. Geng, R. Krasny, G. W. Wei, M. J. Holst, J. A. McCammon and N. A. Baker, Improvements to the APBS biomolecular solvation software suite, *Protein science : a publication of the Protein Society*, **2018**, 27, 10.1002/pro.3280.

[5] S. Kozak, L. Lercher, M. N. Karanth, R. Meijers, T. Carlomagno and S. Boivin, Optimization of protein samples for NMR using thermal shift assays, *Journal of biomolecular NMR*, **2016**, 64, 10.1007/s10858-016-0027-z.

[6] T. C. Miller, B. Simon, V. Rybin, H. Grotsch, S. Curtet, S. Khochbin, T. Carlomagno and C. W. Muller, A bromodomain-DNA interaction facilitates acetylation-dependent bivalent nucleosome recognition by the BET protein BRDT, *Nature communications*, **2016**, 7, 10.1038/ncomms13855.

[7] R. B. Kapust and D. S. Waugh, Escherichia coli maltose-binding protein is uncommonly effective at promoting the solubility of polypeptides to which it is fused, *Protein science : a publication of the Protein Society*, **1999**, 8, 10.1110/ps.8.8.1668.

WILEY-VCH
